# Application of Zebrafish as a Model for Anti-Cancer Activity Evaluation and Toxicity Testing of Natural Products

**DOI:** 10.3390/ph16060827

**Published:** 2023-06-01

**Authors:** Yifan Shen, Ruilong Sheng, Ruihua Guo

**Affiliations:** 1 College of Food Science and Technology, Shanghai Ocean University, Shanghai 201306, China; yifanshen2001@163.com; 2 CQM-Centro de Química da Madeira, Campus da Penteada, Universidade da Madeira, 9000-390 Funchal, Portugal; ruilong.sheng@staff.uma.pt; 3 Shanghai Engineering Research Center of Aquatic-Product Processing & Preservation, Shanghai 201306, China; 4 Laboratory of Quality and Safety Risk Assessment for Aquatic Products on Storage and Preservation (Shanghai), Ministry of Agriculture, Shanghai 201306, China

**Keywords:** zebrafish, natural products, anti-cancer, anti-angiogenesis, toxicity

## Abstract

Developing natural product-based anti-cancer drugs/agents is a promising way to overcome the serious side effects and toxicity of traditional chemotherapeutics for cancer treatment. However, rapid assessment of the in vivo anti-cancer activities of natural products is a challenge. Alternatively, zebrafish are useful model organisms and perform well in addressing this challenging issue. Nowadays, a growing number of studies have utilized zebrafish models to evaluate the in vivo activities of natural compounds. Herein, we reviewed the application of zebrafish models for evaluating the anti-cancer activity and toxicity of natural products over the past years, summarized its process and benefits, and provided future outlooks for the development of natural product-based anti-cancer drugs.

## 1. Introduction

Cancer is one of the leading global public health problems with high mortality rates. In 2022, it was estimated that there were approximately 1.9 million new cases of cancer and 600 thousand deaths from cancer in the United States, of which breast cancer had the highest incidence rate, and lung cancer was the leading cause of death, with approximately 350 people succumbing to it daily [1]. Influenced by the increase in the adult population size, the intensification of the aging population [2], and other social or medical problems, the data above will continue to increase. As a result, tackling cancer has become a major issue for mankind. With the development of biology, immunology, and other disciplines, as well as advances in modern medical treatments, the cure for cancer is gradually achievable [3]. Currently, chemotherapeutic drugs, including cytotoxic drugs, hormonal drugs, regulators of biological reactions, monoclonal antibodies, adjuvants, etc. are implemented as the main approaches toward cancer treatment [4]. Although a number of significant advances were achieved in current treatments, it has been reported that chemotherapy could produce many side effects and needs to be further optimized [3].

Since ancient times, natural products have been used as traditional medicines to treat different diseases because they are inexpensive, accessible, acceptable, and easy-to-apply therapeutic approaches with relatively low toxicity and fewer side effects. Previous research disclosed that natural products could treat cancer through various mechanisms, such as alternating cancer initiation, development, and progression; interrupting cell differentiation, proliferation, and angiogenesis; and inducing cellular apoptosis and inhibiting metastasis [5]. It has also revealed that natural products have the potential for cancer chemoprevention [6] and might be used as multi-drug resistance (MDR) modulators [7]. Research statistics showed that from 1981 to 2019 (as of 30 September 2019), a total of 1881 new medicines were approved worldwide, of which approximately 23.5% were derived from natural products and their derivatives, including 71 natural products (comprising 3.8% of total medicine number); 14 plant medicines (0.8%); and 356 natural product derivatives (18.9%) [8]. Natural products are, therefore, one of the possible sources of anti-cancer drugs. Most natural products, however, have complex components and structures and multiple biotargets and synergistic effects, which complicates the assessment and identification of the active molecular ingredients that may be responsible for their effectiveness [9]. Thus, it is highly desirable to develop reliable and efficient methods for testing the bioactivity of natural products, screening potential anti-cancer components as well as evaluating their efficacy and toxicity.

The zebrafish (*Danio rerio*) is used extensively in research on human disease models, drug screening, drug toxicity and safety assessments, and has many unique benefits. First, the zebrafish genome and human genome share more than 70% similarity, and over 80% of human disease-related genes can be found in the zebrafish genome [10]; moreover, the protein and disease processes are conservative between humans and zebrafish. This means that drugs which are active in humans are often efficacious for zebrafish as well as have the same target [11]. Second, importantly, the zebrafish’s embryos and larvae are transparent, which allows scientists to directly observe the entire process of embryogenesis and also permits the use of optical instrumentation to obtain in vivo cellular and subcellular imaging [12]. Furthermore, the small size, external fertilization, rapid development, high reproduction rate, and low cost of maintenance make the zebrafish a popular animal model [13].

A zebrafish model is a useful tool for screening and developing anti-cancer drugs from natural products. The bioactivity of natural products can be determined by means of high throughput phenotypic screening of live zebrafish [14]. Moreover, the zebrafish model can be used to detect in vivo drug toxicity for preclinical assessment of safety [15], and can be used to comprehensively assess the effectiveness of anti-cancer drugs [16]. Zebrafish have been extensively used in all aspects as an important tool for the research and development of new drugs. Herein, we mainly focus on natural product detection, cancer, and toxicity, and summarize the application of zebrafish as a model for assessing the anti-cancer effect of natural products and detecting the toxicity of natural products, which might provide the foundation for the follow-up investigation of natural products.

## 2. Evaluation of the Anti-Cancer Effect of Natural Products Using Zebrafish Models

Zebrafish models provide great convenience for the molecular investigation of numerous human diseases. Among them, cancer came to be one of the most extensively studied diseases using zebrafish as a model organism [17]. Zebrafish models can be roughly classified into three categories depending on the modeling method. They are as follows: 1. chemical carcinogen-induced zebrafish models; 2. genetically engineered zebrafish models (including transgenic models (Tg), mutant models, and a combination of both); and 3. human cancer cell zebrafish xenograft models [18,19,20,21]. Moreover, for evaluating the bioactivity and toxicity of natural products, the most commonly used zebrafish models are transgenic zebrafish models and human cancer cell zebrafish xenograft models.

### 2.1. Chemical Carcinogen-Induced Zebrafish Models

At the beginning of the establishment of zebrafish models, chemical carcinogens were often utilized to induce tumors. The most common method to promote induction is to bring the carcinogen into contact with zebrafish through water (or diet routes), to induce the carcinogenesis of zebrafish. This method is effective because zebrafish are sensitive to carcinogens and prone to generate tumors [19,22].

Spitsbergen et al., (2000) exposed zebrafish larvae after 3 weeks of mating to *N*-methyl-*N*′-nitro-*N*-nitrosoguanidine (MNNG), a *N*-nitroso compound with strong carcinogenicity to vertebrates, by bath exposure to obtain zebrafish with tumors. After 6 to 12 months, tumors were found mainly in the blood vessels and testes (9% hemangioma or angiosarcoma and 10% seminoma) [23]. Within the same year, they exposed the zebrafish larvae to 7,12-dimethylbenz[*a*]anthracene (DMBA), a potent carcinogenic polyaromatic cyclic hydrocarbon, in the same way to obtain the zebrafish with tumors. Unlike the former study, the liver and gills of zebrafish became the most important tumor target organs (30% hepatic neoplasia and 20% chordoma or chondrosarcoma) [24].

Furthermore, the alkylating-agent ethyl nitrourea (ENU) was used to induce tumors in zebrafish. Beckwith et al., (2000) obtained the zebrafish model by immersing the male zebrafish from 7 to 9 months old in a certain concentration of *N*-ethyl-*N*-nitrosourea (three times every 72 h, one hour each time) at room temperature (22 °C to 23 °C). After 10 to 12 months, it was found that all zebrafish had epidermal papilloma [25].

*N*-nitrosodimethylamine (NDMA) is also a commonly used tumor-inducer for fish. Mizgireuv et al., (2004) exposed zebrafish from 7 to 9 weeks old to a certain concentration of *N*-nitrosodimethylamine aqueous solution for 8 weeks to obtain zebrafish tumor models. After 24 weeks, all tumors were found in the liver, and the incidence rate of tumors was 7.7%; this figure reached 10.3% in the 36th week [26].

Although the above research showed that it seemed feasible to use chemical carcinogens to generate zebrafish cancer models and it was simple in experiments, this method has great defects. First, this method takes a long time to reach completion. After the exposure of zebrafish to carcinogens, it usually takes half a year to one year to produce tumors. Second, the rate of inducing zebrafish to produce tumors by carcinogens is not high, with generally less than a 30% induction rate. In addition, the location of induced tumors varies greatly and cannot be controlled [27]. At the same time, researchers inevitably come into contact with highly toxic carcinogens when using this method, which is a threat to the researchers’ health. Therefore, in recent studies, this method is rarely used to construct zebrafish cancer models for evaluating the anti-cancer activity of natural products.

### 2.2. Genetically Engineered Zebrafish Models

With the developments in molecular biology, especially the technological progress at the biomolecular and gene level, the method of building zebrafish models has gradually changed from chemical carcinogen-induction to genetic engineering. This kind of model is usually obtained by using different forward and reverse genetic methods in the whole zebrafish organism or specific organs, tissues, and glands of zebrafish, combined with new DNA recombination, mutation, and genetic operation technology [28].

The transgenic zebrafish is the most common genetically engineered zebrafish model. The embryos of this kind of zebrafish model are often used to evaluate the anti-angiogenic activity of natural products. Angiogenesis is a hallmark of over 50 different disease states, while its dysfunction is implicated in multiple disorders, including cancer [29]. Angiogenesis is a necessary process of tumor growth, invasion, and metastasis [30]. The free diffusion of oxygen, which is needed for tumor growth, happens only in the capillary terminal. Therefore, the induction of new blood vessels is a necessary condition for tumors to recruit oxygen and nutrients and disseminate to distant sites, and inhibiting abnormal angiogenesis can prevent tumors from growing [31]. Hence, the use of natural products to inhibit angiogenesis is a focus of cancer therapy.

In the study of using transgenic zebrafish to evaluate the anti-angiogenic activity of natural products, these transgenic zebrafish can usually express the enhanced green fluorescent protein (EGFP) or the green fluorescent protein (GFP) in vivo. The most common type is the Tg (fli1:EGFP) (also named Tg (fli1:EGFP)y1, Tg (fli1a:EGFP), or Tg (fli1a:EGFP)y1, which are synonyms) [32,33] line transgenic zebrafish. The Friend leukemia integration 1 (fli1) is a gene expressed in vascular endothelial cells. The fli1:EGFP recombinant gene sequence was inserted into the genome of wild-type transgenic zebrafish. Under the regulation of the fli1 promoter, the gene was transcribed and translated, and the green fluorescent protein (EGFP) was synthesized, so the blood vessels of zebrafish were labeled, which enabled researchers to observe the angiogenesis of zebrafish directly [34].

**Figure 1:**
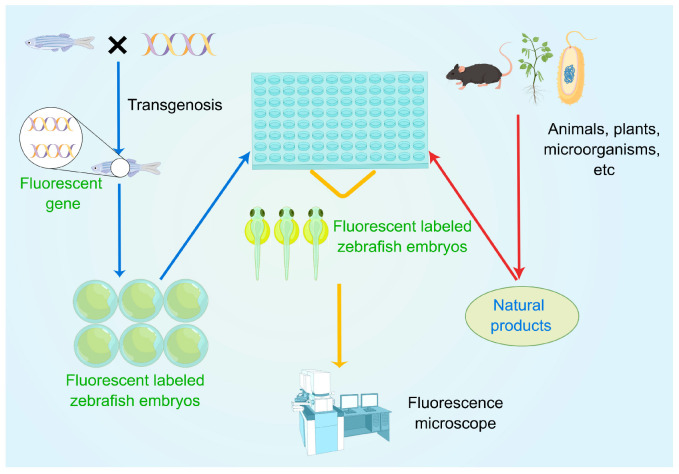
Evaluation of anti-angiogenic activity of natural drugs by transgenic zebrafish models.

**Figure 2:**
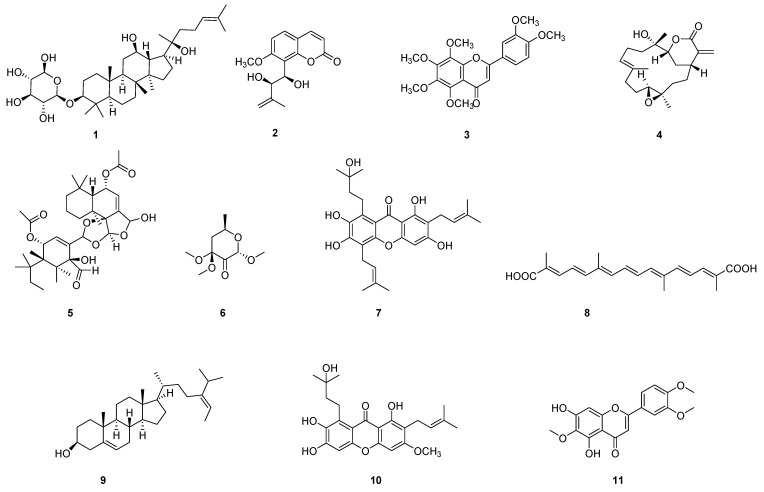
Chemical structures of natural compounds (**1**–**11**) evaluated in zebrafish models.

**Figure 3:**
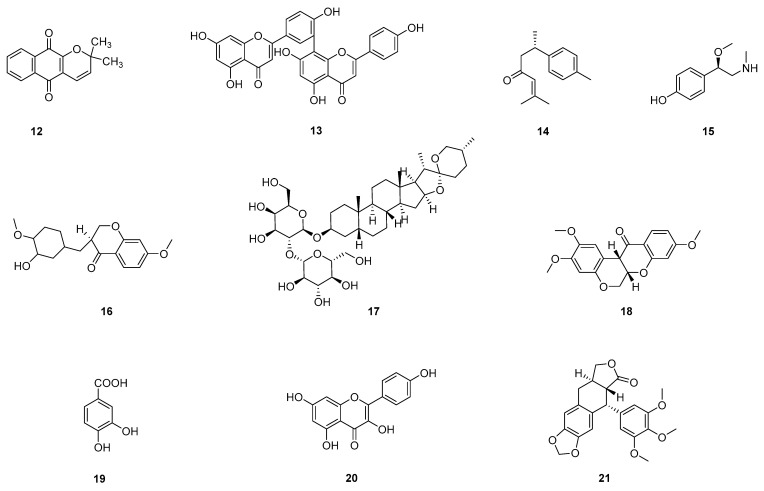
Chemical structures of natural compounds (**12**–**21**) evaluated in zebrafish models.

The process of evaluating the anti-angiogenic activity of natural products using the transgenic zebrafish models is shown in Figure 1. Herein, embryos were first treated with the target natural product under different concentrations, accompanied by a vehicle control and a positive control. After a waiting period (usually 48 or 72 hpf (hour post-fertilization), since the blood vessels could be observed at these times), the zebrafish were anesthetized, fixed, and observed under a fluorescent microscope. In this case, inter-segmental blood vessels (ISVs), sub-intestinal vessels (SIVs), and dorsal longitudinal anastomotic blood vessels (DLAVs) were considered as angiogenic blood vessels [35]. On this basis, the lengths of ISVs, SIVs, or DLAVs were often used as an evaluation index to calculate the inhibition rate and evaluate the anti-angiogenic activity of natural products. Much of the work used a transgenic zebrafish model to evaluate the anti-angiogenic activity of natural products. We classified and introduced these studies based on different recombined genes. Table 1 and Table 2 show the collection of these compounds/extracts. The structures of some compounds (**1**–**21**) can be found in Figure 2 and Figure 3.

#### 2.2.1. Tg (fli1:EGFP) Transgenic Zebrafish Models

Liman et al., (2019) investigated the anti-angiogenic activity of ginsenoside Rh2 (G-Rh2) (**1**) isolated from red ginseng (*Panax ginseng* C. A. Mey.) in zebrafish transgenic for Tg (fli1:EGFP) [36]. The result showed that ginsenoside Rh2 could inhibit the ISVs’ growth in the range from 42.43 to 84.85 μM in a dose-dependent manner, and the ISVs’ growth nearly ceased when the concentration was above 84.85 μM.

Anti-angiogenic activity of murrangatin (**2**), a plant extract isolated from *Murraya alata* Drake, was evaluated by Long et al., (2018) by observing the growth of SIVs in Tg (fli1:EGFP) transgenic zebrafish [37]. The results showed that murrangatin could inhibit the growth of zebrafish SIVs in a dose-dependent manner (10, 50, or 100 μM) and completely block the formation of SIVs at 100 μM.

Lam et al., (2011) used Tg (fli1:EGFP) transgenic zebrafish to assess the anti-angiogenic activity of nobiletin (**3**), a polymethoxylated flavonoid extracted from *Citrus depressa* Hayata [38]. Accordingly, nobiletin could inhibit ISVs in a dose- and time-dependent manner. The inhibition of ISVs by nobiletin was more pronounced at higher concentrations (30–100 μM) and was most pronounced at 24–48 hpf.

The anti-angiogenic activity of fucoidan, a class of sulfated polysaccharides derived from *Fucus vesiculosus*, was examined in Tg (fli1:EGFP) transgenic zebrafish models. Bae et al., (2020) found that the DLAVs, ISVs, and DA of zebrafish treated with fucoidan (300 μg/mL) derived from *F. vesiculosus* were inhibited, and the angiogenesis-related genes in fucoidan-treated zebrafish were repressed substantially [39]. Hsu et al., (2020) also utilized the Tg (fli1:EGFP) transgenic zebrafish model to evaluate the anti-angiogenic activity of fucoidan isolated from *Laminaria japonica* J.E.Areschoug [40]. The results showed that 0.1, 1, and 2 mg/mL fucoidan inhibited the trunk vasculature of embryos by 20%, 50%, and 75%, respectively, and 4 mg/mL fucoidan was fatal to zebrafish embryos.

Additionally, the anti-angiogenic effect of sinularin (**4**) isolated from *Sinularia flexibilis* was tested by Hsu et al., (2022) in Tg (fli1:EGFP) transgenic zebrafish. The mean fluorescence intensity of zebrafish ISVs was used as an assessment index. The results showed that sinularin (5 and 10 μM) was able to inhibit the development of ISVs in zebrafish larvae [29].

Pan et al., (2016) evaluated the anti-angiogenic activity of capsicodendrin (**5**) (CPCD) isolated from *Cinnamosma macrocarpa* H.Perrier using Tg (fli1:EGFP) transgenetic zebrafish embryos [41]. Neovascularization of ISVs in capsicodendrin-treated (2 μM) zebrafish was remarkably diminished in the first 24 hpf. Furthermore, by 48 hpf, overt defects in angiogenesis could be observed, including abnormal SIV formation, a significant reduction in SIV sprouts, and a lack of DLAVs. Consequently, CPCD had a potentially good anti-angiogenic effect.

Hsi et al., (2022) studied the anti-angiogenic activity of penisterine C (**6**) isolated from Marine Algicolous *Penicillium sumatraense* SC29 by Tg (fli1:EGFP) transgenic zebrafish embryos [42]. Penisterine C was found to have 54% and 37% levels of inhibition on ISVs and DLAVs, both types of blood vessels, at concentrations of 10.2 and 20.4 µM, respectively, indicating that penisterine C exhibited anti-angiogenic activity.

Some xanthone derivatives were also found to exhibit anti-cancer properties. Recently, Zhao et al., (2022) discovered the anti-angiogenic activity of xipsxanthone H (**7**) isolated from *Garcinia xishuanbannaensis* Y.H. Li using Tg (fli1:EGFP) transgenic zebrafish models [43]. Xipsxanthone H showed good anti-angiogenic activity, which restrained angiogenesis in a dose-dependent manner, with inhibition rates of 26.4% at 12.5 µM, 37.9% at 25 µM, and 49.6% at 50 µM.

The methanol extract of *Moricandia sinaica* (Boiss.) Boiss.’s leaves was reported by Farooq et al., (2020) to have good anti-angiogenic activity [35]. These researchers found that the methanol extract of *M. sinaica*’s leaves could inhibit angiogenesis in Tg (fli1:EGFP) transgenic zebrafish in a dose-dependent manner. Furthermore, at 40 μM, the crude methanol extract of *M*. *sinaica*’s leaves inhibited 70% of ISVs and DLAVs and 100% of SIVs. Nevertheless, the bioactive chemical component is still not isolated from *M. sinaica*.

In addition, Zhao et al., (2021) investigated the anti-angiogenic activity of crocetin (**8**) isolated from saffron (*Crocus sativus* L.) using Tg (fli1:EGFP) transgenic zebrafish embryos [44]. Crocetin was shown to inhibit the area of SIVs in a dose-dependent manner (5, 10, and 20 μM), and the rate of inhibition reached approximately 50% at 20 μM, indicating its anti-angiogenic activity.

A sterol compound was also found to have anti-angiogenic activity. Bae et al., (2020) used Tg (fli1:EGFP) transgenic zebrafish to evaluate the anti-angiogenic activity of fucosterol (**9**) extracted from brown algae (*Sargassum fusiforme* (Harv.) Setch.) [45]. It is indicated that the formation of ISVs, DLAVs, and part of the DA of the fucosterol-treated zebrafish were interrupted, meaning that fucosterol (40, 60, and 100 μM) could inhibit angiogenesis.

Furthermore, Deshmukh et al., (2023) found that the extract of *Lepista nuda* (Bull.) Cooke had good anti-angiogenic activity using Tg (fli1:EGFP) transgenic zebrafish embryos [46]. The result demonstrated that the crude extract of *L. nuda* affected the ISVs’ development of Tg (fli1:EGFP) transgenic zebrafish embryos in a dose-dependent manner (5, 10, 50, and 100 µg/mL), causing shorter ISVs relative to untreated embryos.

Like xipsxanthone H (**7**), another xanthone compound, cratoxylumxanthone C (**10**) isolated from *Cratoxylum cochinchinense* (Lour.) Blume, also exhibited anti-angiogenic effects in the Tg (fli1: EGFP) transgenic zebrafish model [47]. In this study, Li et al., (2022) found that the ISVs’ length of the zebrafish decreased from 2560 to 1586 µm with increasing concentrations of cratoxylumxanthone C (5, 10, and 20 µM), indicating that cratoxylumxanthone C had a blockage effect on the blood vessels of zebrafish.

Moreover, Lee et al., (2020) investigated the anti-angiogenic activity of eupatilin (**11**) isolated from *Artemisia asiatica* Nakai ex Pamp. using Tg (fli1:EGFP) transgenic zebrafish embryos [48]. As a result, DLAVs and ISVs were completely destroyed by eupatilin treatment (10, 25, and 50 μM). All these are indicative of the superior anti-angiogenic activity of eupatilin.

Ma et al., (2022) found that the extract of *Synsepalum dulcificum* (Schumach. & Thonn.) Daniell (Miracle berry)’s leaves had good anti-angiogenic activity with the Tg (fli1:EGFP) transgenic zebrafish model [49]. The area of the SIVs’ coverage in embryo yolk was used as an assessment index. The results showed that the MBL extract (10, 25, and 50 μg/mL) could inhibit the formation of zebrafish SIVs. Treatment of embryos with 10 μg/mL extract resulted in a 50.05% inhibition rate of SIVs. The data increased to 62.78% and 79.21% when the concentration was increased to 25 μg/mL and 50 μg/mL, respectively.

Li et al., (2016) used the Tg (fli1:EGFP) transgenic zebrafish model to evaluate the anti-angiogenic activity of proanthocyanidins isolated from *Choerospondias axillaris* (Roxb.) B.L.Burtt & A.W.Hill peels [50]. In their study, the growth of SIVs of zebrafish treated with proanthocyanidins (12.5, 25, and 50 μM) were inhibited in a dose-dependent manner compared with the control group. At the concentration of 50 μM, the inhibition rate was about 50%, indicating the anti-angiogenic activity of proanthocyanidins.

After in vitro screening of 50,000 compounds, Garkavtsev et al., (2011) also verified the anti-angiogenic activity of dehydro-α-lapachone (DAL), a natural product from the *Tabebuia avellanedae* Lorentz ex Griseb. tree (**12**) using Tg (fli1:EGFP) zebrafish models [51]. In comparison with the control, it was found that zebrafish embryos treated with DAL (5 μM) could not form vessel branches and have defects in anastomosis and plexus formation.

Similarly, Li et al., (2017) used the Tg (fli1:EGFP) transgenic zebrafish model to assess the anti-angiogenic activity of amentoflavone (**13**) extracted from *Garcinia xanthochymus* Hook.f. ex T.Anderson [52]. The result showed that the length of SIVs from amentoflavone-treated zebrafish (5, 10, and 20 µM) was decreased in a dose-dependent manner. At 20 µM, the length of SIVs shortened by almost one-third and the expressions of Angpt2 and Tie2 genes were downregulated, which demonstrated the anti-angiogenic effects of amentoflavone.

Turmerones are sesquiterpene compounds isolated from turmeric (*Curcuma longa* L.). In line with this, Yue et al., (2015) investigated the anti-angiogenic activity of (S)-aromatic (Ar)-turmerone (**14**) isolated from the rhizome of *C. longa.* in the Tg (fli1:EGFP) transgenic zebrafish model [53]. Their study showed that (S)-Ar-turmerone (12.5–25 µg/mL) was found to decrease the length of SIVs in a dose-dependent manner. At 5 µM, the length of SIVs shortened by almost half. Moreover, several angiogenic genes (Ang-1, Ang-2, Tie-1, and Tie-2) were downregulated in zebrafish treated with (S)-Ar-turmerone. Accordingly, (S)-Ar-turmerone has good anti-cancer activity.

Kim et al., (2015) used Tg (fli1:EGFP) transgenic zebrafish embryos to investigate the anti-angiogenic activity of *R*-(-)-β-*O*-Methylsynephrine (OMe-Syn) (**15**) isolated from plants in the family *Rutaceae* (Juss.) [54]. As a result, OMe-Syn (2.5 μM or 5.0 μM) inhibited the growth of zebrafish ISVs in a dose-dependent manner. Furthermore, the number of complete ISVs in zebrafish treated with OMe-Syn at 5 µM decreased by about two-thirds compared with the control group, showing the anti-angiogenic activity of OMe-Syn.

Chen et al., (2020) verified the anti-angiogenic activity of deoxysappanone B 7.4′-dimethyl ether (**16**) isolated from *Biancaea sappan* (L.) Tod. using Tg (fli1:EGFP) zebrafish models [55]. They found that, at 48 hpf, the formation of ISVs was inhibited by deoxysappanone B 7.4′-dimethyl ether at 5 μM and the inhibition rate was as high as 99.64%. The high inhibition rate illustrates that deoxysappanone B 7.4′-dimethyl ether has the potential to become an efficient anti-angiogenic agent.

In addition to the saponin compound ginsenoside Rh2 (**1**), another saponin timosaponin AIII (Timo AIII) (**17**), also exhibited anti-angiogenic activity. Zhou et al., (2019) evaluated the anti-angiogenic activity of Timo AIII derived from the traditional Chinese herb *Anemarrhena asphodeloides* Bunge using Tg (fli1:EGFP) transgenic zebrafish [56]. Timo AIII was found to increase the number of defective ISVs and decrease the total area of SIVs in a dose-dependent manner (0.5, 1, 2, and 3 μM). Moreover, the number of intact ISVs was reduced by one-third and the area of SIVs reduced by 40% at a concentration of 3 μM, which demonstrated that Timo AIII had anti-angiogenic activity.

Furthermore, Chen et al., (2018) used Tg (fli1:EGFP) transgenic zebrafish embryos to verify the anti-angiogenic activity of mundoserone (**18**), a natural product isolated from *Pongamia pinnata* (L.) Pierre [57]. At 48 hpf, the morphology of the ISVs was visually assessed. The results showed that the 10 µM mundoserone had an inhibition rate of 73.55% on the ISVs of zebrafish.

Lastly, Hu et al., (2018) evaluated the anti-angiogenic activity of protocatechuic acid (**19**) derived from the sclerotium of *Pleurotus tuber-regium* (Fries) Sing. and *Agrocybe aegerita* (Aa, V. Brig.) Singer using Tg (fli1:EGFP) transgenic zebrafish models [58]. As can be seen from the study results, protocatechuic acid inhibited the SIVs of zebrafish embryos, and the inhibition rate reached 20% at a concentration of 25 µM.

**Table 1:** Anti-angiogenic activity of natural products in Tg (fli1: EGFP) transgenic zebrafish models.

Year	Compound/Extract	Source	Effective Concentration	Blood Vessel	Positive Control	Growing Stage of Zebrafish
2020 [35]	Leaves extract	*Moricandia sinaica* (Boiss.) Boiss.	40 mg/mL	ISVs, DLAVs, SIVs.	-	48 and 72 hpf
2023 [46]	extract	*Lepista nuda* (Bull.) Cooke	-	ISVs	-	24 hpf
2022 [49]	Leaves extract	*Synsepalum dulcificum* (Schumach. & Thonn.) Daniell (Miracle berry) leaves	-	SIVs	-	72 hpf
2016 [50]	Proanthocyanidins	*Choerospondias axillaris* (Roxb.) B.L.Burtt & A.W.Hillpeels	-	SIVs	SU5416	72 hpf
2020 [39]	Fucoidan	*Fucus vesiculosus*	300 µg/mL	ISVs, DLAVs, DA.	-	48 hpf
2020 [40]	Fucoidan	*Laminaria japonica* J.E.Areschoug	-	the trunk, vasculature.	-	48 hpf
2019 [36]	Ginsenoside Rh2 (**1**)	red ginseng (*Panax ginseng* C. A. Mey.)	-	ISVs	SU5416	48 hpf
2018 [37]	Murrangatin (**2**)	*Murraya alata* Drake	-	SIVs	-	72 hpf
2011 [38]	Nobiletin (**3**)	*Citrus depressa* Hayata	-	ISVs	VEGFR inhibitor II	32 hpf
2022 [29]	Sinularin (**4**)	soft coral (*Sinularia flexibilis*)	-	ISVs	-	72 hpf
2016 [41]	Capsicodendrin (**5**)	*Cinnamosma macrocarpa* H.Perrier	2 μM	DLAVs, SIVs.	-	48 hpf
2022 [42]	Penisterine C (**6**)	Algicolous *Penicillium sumatraense* SC29	-	ISVs, DLAVs.	Sorafenib	96 hpf
2022 [43]	Xipsxanthone H (**7**)	*Garcinia xishuanbannaensis* Y.H. Li	-	ISVs	Sunitinib malate	48 hpf
2021 [44]	Crocetin (**8**)	saffron (*Crocus sativus* L.)	-	SIVs	VEGFR tyrosine kinase inhibitor II	72 hpf
2020 [45]	Fucosterol (**9**)	brown algae (*Sargassum fusiforme* (Harv.) Setch.)	-	ISVs, DLAVs, DA.	-	24 hpf
2022 [47]	Cratoxylumxanthone C (**10**)	*Cratoxylum cochinchinense* (Lour.) Blume	-	ISVs	Sunitinib malate	54 hpf
2020 [48]	Eupatilin (**11**)	*Artemisia asiatica* Nakai ex Pamp.	100 μM	DLAVs, ISVs, DA.	-	48 hpf
2011 [51]	Dehydro-α-lapachone (**12**)	*Tabebuia avellanedae* Lorentz ex Griseb. tree	5 μM	ISVs	-	48 hpf
2017 [52]	Amentoflavone (**13**)	*Garcinia xanthochymus* Hook.f. ex T.Anderson	-	SIVs	Eriocalyxin B	72 hpf
2015 [53]	Aromatic turmerone (**14**)	*Curcuma longa L.* (Turmeric)	-	SIVs	-	48 hpf
2015 [54]	*R*-(-)-β-*O*-Methylsynephrine (**15**)	*Rutaceae* (Juss.) family	-	ISVs	-	30 hpf
2020 [55]	Deoxysappanone B 7.4′-dimethyl ether (**16**)	*Biancaea sappan* (L.) Tod.	5 μM	ISVs	PTK787	48 hpf
2019 [56]	Timosaponin AIII (**17**)	*Anemarrhena asphodeloides* Bunge	-	ISVs, SIVs.	-	36 hpf
2018 [57]	Mundoserone (**18**)	*Pongamia pinnata* (L.) Pierre	-	ISVs	PTK787	24 and 48 hpf
2018 [58]	Protocatechuic acid (**19**)	*Pleurotus tuberregium* (Fries) Sing and *Agrocybe aegerita* (Aa, V. Brig.) Singer	25 µM	SIVs	SU5416	48 and 72 hpf

#### 2.2.2. Other Transgenic Zebrafish Models

The endothelial cell-specific transgenic zebrafish line Tg (kdrl: GRCFP)^zn1^ (the same as Tg (VEGFR2:GFP)) can directly label zebrafish vascular endothelial cells. Among them, GFP (green fluorescent protein) is controlled by the promoter of vascular endothelial growth factor receptor 2 (VEGFR2, also known as kdrl or flk, Entrez gene ID: 796537). Liang et al., (2015) used Tg (kdrl:GRCFP)^zn1^ transgenic zebrafish to assess the anti-angiogenic effect of kaempferol (**20**) isolated from *Dysosma versipellis* (Hance) M.Cheng [30]. Zebrafish embryos are supplemented with kaempferol, and the ISVs’ length was used as an assessment index at 48 hpf. The results demonstrated that ISVs from kaempferol treatment groups were shorter in duration compared to the control group, indicating its anti-angiogenic activity.

Wang et al., (2015) discovered that deoxypodophylloxin (DPT) (**21**) isolated from *Anthriscus sylvestris* (L.) Hoffm. had the anti-angiogenic effect in vivo using Tg (VEGFR2:GFP) transgenic zebrafish [34]. It was found that the ISV length of DPT-treated embryos was shorter at 24 hpf compared to the control group, indicating that zebrafish blood vessel growth was inhibited.

Moreover, the red fluorescent transgenic zebrafish model Tg (flk:mCherry) is a transgenic zebrafish with the flk promoter directing the expression of red fluorescent protein variant mCherry, which was also employed to evaluate natural products. Zhang et al., (2017) assessed the anti-angiogenic activity of *Euphorbia pekinensis* Rupr. (EP) water extract in the Tg (flk:mCherry) zebrafish model (with red fluorescence in blood vessels) [59]. It was found that the number of intact blood vessels decreased from 26.00 ± 1.29 to 20.80 ± 1.75 as the concentration of EP extract increased from 100 µg/mL to 250 µg/mL.

In addition to this model, the Tg (flk1:EGFP) is a transgenic zebrafish line with the flk1 (VEGFR-2) promoter directing EGFP expression. Thus, it is highly appropriate for the evaluation of anti-angiogenesis drugs. Zhong et al., (2017) obtained an extract from *Ilex kudingcha* C.J. Tseng and evaluated its anti-angiogenic activity with the Tg (flk1:EGFP) transgenic zebrafish model [60]. The results demonstrated that different concentrations of kudingcha extracts could inhibit ISV growth in zebrafish embryos.

**Table 2:** Anti-angiogenic activity of natural products in other transgenic zebrafish models.

Year	Compound/Extract	Source	Zebrafish Type	Effective Concentration	Blood Vessel	Positive Control	Growing Stage of Zebrafish
2017 [59]	water extract	*Euphorbia pekinensis* Rupr.	Tg (flk:mCherry)	-	ISVs	PTK787	72 hpf
2017 [60]	kudingcha extract	*Ilex kudingcha* C.J. Tseng	Tg (flk1:EGFP)	-	ISVs	-	52 hpf
2015 [30]	Kaempferol (**20**)	*Dysosma versipellis* (Hance) M.Cheng	Tg (kdrl:GRCFP)^zn1^	40 µM	ISVs	-	48 hpf
2015 [34]	Deoxypodophyllotoxin (**21**)	*Anthriscus sylvestris* (L.) Hoffm.	Tg (VEGFR2:GFP)	50 nM	ISVs	-	50 hpf

### 2.3. Human Cancer Cell Zebrafish Xenograft Models

Drug development is a lengthy process, and drugs must undergo preclinical and clinical studies before they can be approved for commercial release. Owing to ethical and practical limitations, current in vivo human somatic cell research is restricted to xenotransplantation [61], the transfer of material isolated from one species into another [62]. As with the mouse xenograft model, the zebrafish xenograft model provides a good pre-clinical animal model. Nearly 20 years have passed since the introduction of the first zebrafish xenograft model in 2005 [63]. During this period, technology has been continually refined and modernized, gradually maturing and being placed in drug research and development.

The mouse xenograft model has long served as a “gold standard” for studying cancer cells in vivo [64]. Nevertheless, scientists have gradually uncovered the benefits of the zebrafish xenograft model, and to some extent, use it for mouse replacement, or use both to assess the anti-cancer activity of the drugs. The zebrafish embryo xenograft model has been the most widely used model in a variety of studies for evaluating natural product activities. The advantages of the zebrafish embryo xenograft model over mice are as follows: (I) the zebrafish model is small in size, low in cost, and easy to maintain; (II) the model has a short reproductive cycle and high egg production (7 days, 200 embryos); (III) the embryos are transparent, allowing direct imaging of development, organogenesis, and progression of cancer xenograft; (IV) there is less demand for human cancer cells in each embryo (50–300 cells); (V) there is a lack of an adaptive immune system and absence of a rejection reaction; and (VI) small bioactive molecules transported by water are permeable, so that the molecules can be put directly into the culture solution for the purpose of treatment [62,63,65,66].

**Figure 4:**
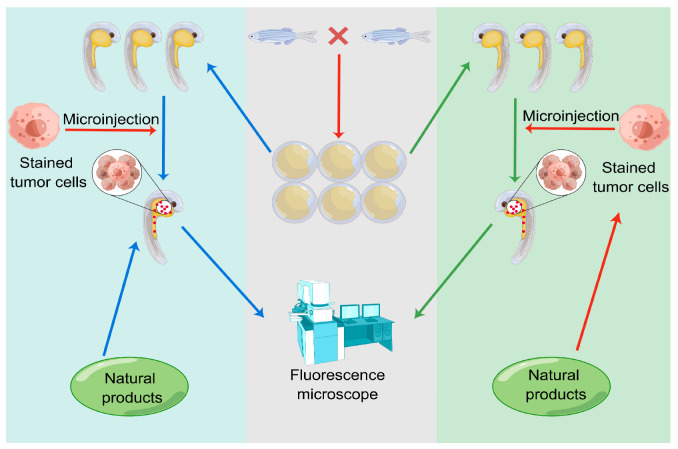
Evaluation of anti-tumor activity of natural drugs through zebrafish xenograft models.

The process of evaluating the anti-cancer activity of natural products using the human cancer cell zebrafish xenograft models is shown in Figure 4. On the left side of Figure 4, human cancer cells were first implanted into zebrafish, and then, the zebrafish were treated with the natural product solution/control solution (treatment before implantation), and finally the results were observed. On the right side of Figure 4, human cancer cells were first treated with the natural product solution/control solution (implantation before treatment), and then implanted into zebrafish, and the results were subsequently observed. The difference here lies in the order between implantation and treatment.

**Figure 5:**
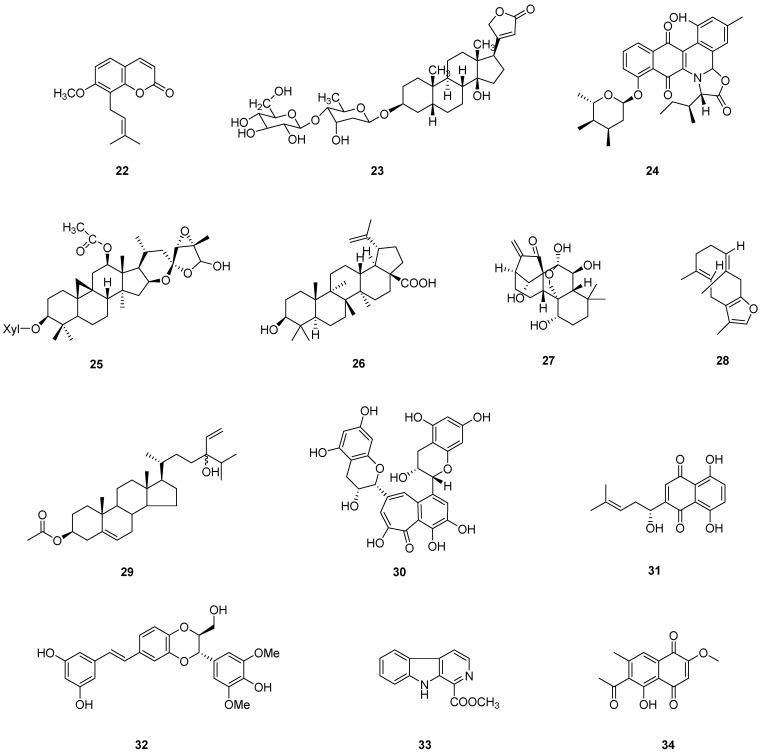
Chemical structures of natural compounds (**22**–**34**) evaluated in zebrafish models.

When establishing a zebrafish xenograft model, the following aspects should be noted. First, the temperature at which the zebrafish embryos are reared. The studies have shown 28 °C to be the optimum developmental temperature for zebrafish embryos, but human cancer cells adapt to proliferating and growing at 37 °C. Thus, it has been experimentally demonstrated that 34 to 35 °C is a compromised temperature condition to allow both xenografted cells and hosts to grow [67]. The next step is to determine the location of the cancer cell injection. Orthotopic xenotransplantation is not always feasible in the zebrafish model, so heterotopic transplantation is used in the majority of cases. The most popular injection site is the yolk sac, primarily due to its large size, ease of operation, and ability to accommodate many cells, and, secondly, because it is a nutritive environment which is conducive to cell proliferation [63,68]. The third point is the age of the zebrafish embryo, with the vast majority of experiments taking zebrafish embryos at 48 hpf because, at this time, the embryo vasculature and organ system gradually start to develop, which will not induce developmental defects. Simultaneously, the embryo fails to produce an immune system, and the yolk sac grows large [63]. Overall, we demonstrated several examples involving the utilization of zebrafish embryo xenograft models to assess the anti-cancer capacity of natural products in recent years. Table 3 shows the collection of these compounds/extracts. The structures of some compounds (**22**–**34**) can be found in Figure 5.

#### 2.3.1. Ovarian Cancer

The anti-angiogenic activity of fucoidan has been examined by Bae et al., (2020) in Tg (fli1:EGFP) transgenic zebrafish models [39]. In the same study, they also used a zebrafish xenograft model to verify the anti-cancer ability of the fucoidan extracted from *F. vesiculosus* on ovarian cancer. They first treated ES2 and OV90 cells with fucoidan and then incubated them with the CM-Dil dye as a cell tracker. The two kinds of cells were injected into the yolk sac of zebrafish by a microinjector. After incubation, fucoidan could gradually inhibit tumor formation in ES2 and OV90 cells, leading to a decrease in tumor size in vivo.

Osthole (**22**) is a natural product isolated from *Angelica archangelica* L., *Angelica pubescens* Maxim., and *Cnidium monnieri* (L.) Cusson. Bae et al. studied the in vivo inhibitory effect of osthole (**22**) on ovarian cancer using a zebrafish xenograft model [68]. They treated embryos with Danieau’s solution of 0.003% phenylthiourea (PTU) to suppress pigmentation and microinjected ES2 cells and OV90 cells stained with CM-Dil dye into the yolk sac of zebrafish embryos. After incubation for 72 h, the tumor formation was decreased to 46.92% and 42.53% in ES2 cells and OV90 cells (20 µM osthole), respectively. Bae et al., (2020) also evaluated the anti-cancer activity of fucosterol (**9**) found in brown algae (*S. fusiforme*) in the same way [45]. The results showed that tumor formation in fucosterol-treated zebrafish (100 µM) was decreased to 58.0% in ES2 cells and to 60.4% in OV90 cells.

Lee et al., (2020) also constructed a zebrafish xenograft model with ES2 cells and OV90 cells to evaluate the anti-cancer activity of eupatilin (**11**) derived from *A. asiatica* [48]. They microinjected the tetramethylrhodamine (TMR) red-labeled ES2 cells and OV90 cells into the zebrafish embryos. After treatment, they found that eupatilin (10, 20, 40, 50, and 100 µg/mL) could inhibit the growth of ES2 cells and OV90 cells. Furthermore, eupatilin at concentrations of 50–100 µg/mL triggered a complete abrogation of tumorigenesis in vivo.

#### 2.3.2. Non-Small Cell Lung Cancer

The anti-non-small cell lung cancer ability of xipsxanthone H (**7**) isolated from *G. xishuanbannaensis* was verified by Zhao et al., (2022) in zebrafish xenograft models [43]. Zebrafish embryos were microinjected with A549 cells stained with CM-Dil dye and treated with different concentrations of xipsxanthone H (6, 30, and 60 µM). Then, the researchers observed the embryos and found that the fluorescence intensity and focus of zebrafish decreased in a dose-dependent manner, indicating that xipsxanthone H inhibited the migration and proliferation of tumor cells in vivo.

Additionally, Li et al., (2022) found that cratoxylumxanthone C (**10**) isolated from *C. cochinchinense* possessed significant anti-cancer activity on non-small cell lung cancer using a zebrafish xenograft model [47]. They labeled A549 cells with CM-Dil dye, and then microinjected the cells into the yolk sac of zebrafish embryos. After treatment with cratoxylumxanthone C (2.5, 5, and 10 µM) for 48 h, the fluorescence intensity was decreased. Therefore, the proliferation and migration of A549 cells were significantly inhibited in vivo.

Similarly, Schneider et al., (2018) studied the lung cancer inhibitory effect of cardenolide glucoevatromonoside (**23**) (GEV) derived from *Digitalis lanata* Ehrh. using the zebrafish xenograft model [67]. They treated A549 cells with GEV (10, 50, and 100 nM) hours and stained A549 cells with CM-Dil dye. Then, they injected A549 cells into the yolk sac of zebrafish and embryos were incubated. As a result, GEV inhibited the development of tumor cells. Moreover, GEV concentrations over 50 nM had a strong growth-inhibitory effect on A549 tumor cells (>40% tumor cell death) in vivo.

#### 2.3.3. Breast Cancer

McKeown et al., (2022) used a zebrafish xenograft model to assess the anti-tumor activity of Jadomycin B (**24**), a secondary metabolite of the oil bacteria *Streptomyces venezuelae* ISP5230 [69]. They injected fluorescently labeled MDA-MB-231 cells into the yolk sac of zebrafish and counted the number of cells in the zebrafish. The results showed that Jadomycin B could inhibit MDA-MB-231 tumor cell numbers in a dose-dependent manner (10, 20, and 40 µM) and the number of tumor cells decreased by 33% at 40 μM.

Wu et al., (2018) discovered that actein (**25**) extracted from the root of *Cimicifuga* species had the anti-cancer effect in vivo by using a zebrafish xenograft model [70]. They microinjected Dil-labeled MDA-MB-231 cells into the zebrafish yolk sac and cultured them in actein solution. Consequently, the MDA-MB-231 cells in zebrafish embryos treated with actein (60 µM) were significantly reduced by 74% when compared with the control group.

In addition to this compound, betulinic acid (**26**) is a pentacyclic triterpene identified from birch (*Betula platyphylla* Sukaczev) bark. Jiao et al., (2019) used a human breast cancer zebrafish xenograft model to assess the anti-cancer activity of betulinic acid [71]. They first labeled MCF-7 cells with Dil, and then microinjected the cells into the perivitelline space of zebrafish embryos. Following this, they added the betulinic acid solution (20 µM–80 µM) to aquaculture water. The tumor inhibition rate was calculated. It was indicated that betulinic acid above 20 µM could significantly inhibit the development of MCF-7 cells in zebrafish.

Hsu et al., (2020) also assessed the anti-cancer ability of fucoidan isolated from *L. japonica* on breast cancer utilizing a zebrafish xenograft model [40]. GFP-expressing MDA-MB-231 cells were treated with fucoidan (2 µg/mL), and were injected into the perivitelline cavity of zebrafish. The results showed that 18% of fucoidan-treated tumor cells exhibited micrometastasis, indicating that fucoidan could inhibit the growth of MDA-MB-231 tumor cells in vivo in zebrafish.

#### 2.3.4. Liver Cancer

Tian et al., (2017) constructed a zebrafish xenograft model to evaluate the anti-cancer activity of oridonin (**27**) obtained from *Rabdosia rubescens* (Henmsl.) H.Hara on liver cancer [72]. They labeled the HepG2-Luciferase cells with Dil die and microinjected them into the perivitelline space of zebrafish embryos. Subsequently, they incubated embryos with oridonin (30 cells per nl). It is found that oridonin could inhibit the development of HepG2-Luciferase cells in vivo.

Zhu et al., (2019) have also constructed a zebrafish xenograft model with HepG2 hepatoma cells to evaluate the anti-cancer activity of furanodiene (**28**) found in *Curcuma longa* L. [73]. They inserted the CM-Dil-labeled cells into the yolk of zebrafish by microneedles and added the treatment solution to the fish water. The results demonstrated that zebrafish treated with furanodiene had survival days that were 1, 2, and 2.67 times longer than those of the control group.

In addition to natural sterols [45], sterol-based derivatives (e.g., ester) were also found to have anti-cancer ability. For instance, Kim et al., (2021) verified the anti-cancer ability of saringosterol acetate (SSA) (**29**) isolated from *Sargassum fusiforme* (Harv.) Setch. on liver cancer utilizing a zebrafish xenograft model [74]. They first injected a certain amount of SSA into zebrafish (2 µg/g or 5 µg/g) and then CM-Dil-labeled Hep3B cells were microinjected into the abdominal cavity of zebrafish. It was found that the survival rate of Hep3B cells in the SSA group decreased, indicating that SSA had an inhibitory effect on zebrafish xenograft hepatocellular carcinoma.

#### 2.3.5. Melanoma

Zhang et al., (2020) used a zebrafish xenograft model to verify the anti-cancer ability of theaflavin (TF) (**30**) extracted from the tea plant species *Camellia sinensis* (L.) Kuntze on melanoma [75]. They labeled A375 cells with CM-Dil and microinjected the cells into the yolk sac of larval zebrafish. After this, zebrafish were treated with different concentrations of TF (0.4, 1.3, and 3.9 µM) and were observed under a fluorescence microscope. The consequences showed that TF inhibited A375 cells in a dose-dependent manner, and the inhibition rate was 1.0% to 46.4% in the range of 0.4 to 3.9 µM.

Cao et al., (2020) constructed a zebrafish xenograft model with A375 and A2058 cells to evaluate the anti-cancer activity of shikonin (**31**) isolated from *Arnebia euchroma* (Royle ex Benth.) I.M.Johnst [76]. CM-Dil labeled A375 and A2058 cells were first treated with different concentrations of shikonin (0.015, 0.0625, and 0.25 µM), and the cells were subsequently observed using fluorescence microscopy. The results showed that red fluorescence in zebrafish yolk was reduced after shikonin treatment in a dose-dependent manner.

#### 2.3.6. Other Malignancies

Chen et al., (2022) constructed a zebrafish xenograft model to evaluate the anti-tumor activity of aiphanol (**32**) extracted from *Smilax glabra* Roxb [77]. They microinjected the human colorectal HCT116 and HT29 cancer cells into the yolk of zebrafish and treated these zebrafish with different concentrations of aiphanol (1.5, 5, and 15 µM). The fluorescence intensity was calculated. Therefore, they found that the number of HCT116 and HT29 cells microinjected in zebrafish treated with aiphanol decreased, indicating that aiphanol had a certain inhibitory effect on colorectal cancer cells.

Furthermore, Lin et al., (2018) used a zebrafish xenograft model to assess the anti-cancer ability of 1-methoxycarbonyl-β-carboline (MCC) (**33**) extracted from *Picrasma quassioides* (D. Don) Benn [78]. DU145 cells were first pre-treated with 50 µM MCC. Following the pre-treatment, the cells were labeled with CM-Dil and microinjected into the yolk sac of 48 hpf zebrafish. The results for this study illustrated that the number of DU145 cells in zebrafish treated with MCC (50 µM) decreased remarkably, indicating its anti-cancer activity.

Theabrownins (TBs) are bioactive polymeric pigments found in dark tea. Jin et al., (2018) studied the inhibitory effect of TBs from the tea plant species *Camellia sinensis* (L.) Kuntze on osteosarcoma U2OS cancer cells using the zebrafish xenograft model [79]. U2OS cells were first treated with TBs of different concentrations in their logarithmic growth phase. Then, the cells were labeled with CM-Dil and microinjected into the yolk sac of zebrafish. The consequences indicated that the inhibition rate of different concentrations of TBs on U2OS cells ranged from 24.6% to 27.3% (100–200 µg/mL).

2-methoxy-6-acetyl-7-methyljuglone (MAM) (**34**) is a naphthoquinone isolated from the *Ventilago denticulata* Willd. or *Rumex japonicus* Houtt. Yua et al., (2020) assessed the anti-cancer ability of 2-methoxy-6-acetyl-7-methyljuglone (MAM) (**34**) on glioblastoma utilizing a zebrafish xenograft model [80]. They microinjected the Dil-labeled U251 cells into the yolk of zebrafish. After injection, zebrafish were treated with MAM of different concentrations (50 and 200 nM). The results demonstrated that MAM could significantly inhibit the development of U251 cells, and the fluorescence area decreased to 30% at 200 nM.

**Table 3:** Application of zebrafish xenograft models to detect the anti-cancer activity of natural products.

Year	Compound/Extract	Source	Cells	Effective Concentration	Type of Cancer	Positive Control	Growing Stage of Zebrafish
2020 [39]	Fucoidan	*F. vesiculosus*	ES2 cells and OV90 cells	-	Ovarian Cancer	-	72 hpf
2020 [68]	Osthole (**22**)	*Angelica archangelica* L., *Angelica pubescens* Maxim., *Cnidium monnieri* (L.) Cusson	ES2 cells and OV90 cells	-	Ovarian Cancer	-	72 hpf
2020 [45]	Fucosterol (**9**)	brown algae (*S. fusiforme*)	ES2 cells and OV90 cells	-	Ovarian Cancer	-	72 hpf
2021 [48]	Eupatilin (**11**)	*A. asiatica*	ES2 cells and OV90 cells	-	Ovarian Cancer	-	48 hpf
2022 [43]	Xipsxanthone H (**7**)	*G. xishuanbannaensis*	A549 cells	-	Non-small cell lung cancer	Etoposide	48 hpf
2022 [47]	Cratoxylumxanthone C (**10**)	*C. cochinchinense*	A549 cells	-	Non-small cell lung cancer	Etoposide	100 hpf
2018 [67]	Cardenolide glucoevatromonoside (**23**)	*Digitalis lanata* Ehrh.	A549 cells	-	Non-small cell lung cancer	-	48 hpf
2020 [40]	Fucoidan	*L. japonica*	MDA-MB-231 cells	2 µg/mL	Breast cancer	-	48 hpf
2022 [69]	Jadomycin B (**24**)	*Streptomyces venezuelae* ISP5230	MDA-MB-231 cells	-	Breast cancer	-	120 hpf
2018 [70]	Actein (**25**)	*Cimicifuga* species	MDA-MB-231 cells	-	Breast cancer	-	168 hpf
2019 [71]	Betulinic acid (**26**)	birch (*Betula platyphylla* Sukaczev) bark	MCF-7 cells	-	Breast cancer	-	72 and 96 hpf
2017 [72]	Oridonin (**27**)	*Rabdosia rubescens* (Henmsl.) H.Hara	HepG2-Luciferase cells	-	Breast cancer	Avastin	222 hpf
2019 [73]	Furanodiene (**28**)	*Curcuma longa* L.	MCF-7 cells	-	Liver cancer	-	96 hpf
2020 [74]	Saringosterol acetate (**29**)	*sargassum fusiforme* (Harv.) Setch.	Hep3B cells	12.5 μg/mL	Liver cancer	-	96 hpf
2020 [75]	Theaflavin (TF) (**30**)	*Camellia sinensis* (L.) *Kuntze*	A375 cells	-	Melanoma	-	72 hpf
2020 [76]	Shikonin (**31**)	*Arnebia euchroma* (Royle ex Benth.) I.M.Johnst.	A375 and A2058 cells	-	Melanoma	Sorafenib	72 and 96 hpf
2022 [77]	Aiphanol (**32**)	*Smilax glabra* Roxb.	HCT116 and HT29 cells	-	Rectal cancer	5-FU	96 hpf
2018 [78]	1-Methoxycarbony-β-carboline (**33**)	*Picrasma quassioides* (D. Don) Bennet	DU145 cells	50 µM	Prostatic cancer	-	72, 144, and 240 hpf
2018 [79]	Theabrownin	*Camellia sinensis* (L.) Kuntze	U2OS cells	-	Osteosarcoma	-	72 hpf
2018 [80]	2-Methoxy-6-acetyl-7-methyljuglone (**34**)	*Ventilago denticulate* Willd., *Rumex japonicus* Houtt.	U251 cells	-	Glioblastoma	Temozolomide	144 hpf

## 3. Toxicity Testing of Natural Products Using Zebrafish Model

Toxicity testing is an important part of drug research and development. About 20 to 50 percent of drugs fail clinically due to having high levels of toxicity. Therefore, it is highly desirable to detect and evaluate drug toxicity before clinical experiments, which might avoid investing large amounts of resources on the drugs terminated because of high toxicity or severe side effects [81]. Toxicology testing was divided into in vivo and in vitro toxicity assays. The zebrafish is a highly attractive animal model for in vivo toxicity testing.

The zebrafish embryo is one of the most commonly used tools when examining in vivo toxicity of natural products. The advantages of zebrafish embryos over traditional rats and mice as useful tools for in vivo toxicity testing are as follows: (I) ease of rearing, small space required, and low maintenance cost; (II) short growth cycle and rapid assessment; (III) transparent and easy to observe; (IV) drugs could be added directly into the water (or culture medium) for absorption; and (V) easy to operate and less demand for the types of drugs [82,83]. These advantages make zebrafish embryos a powerful tool for toxicity testing.

**Figure 6:**
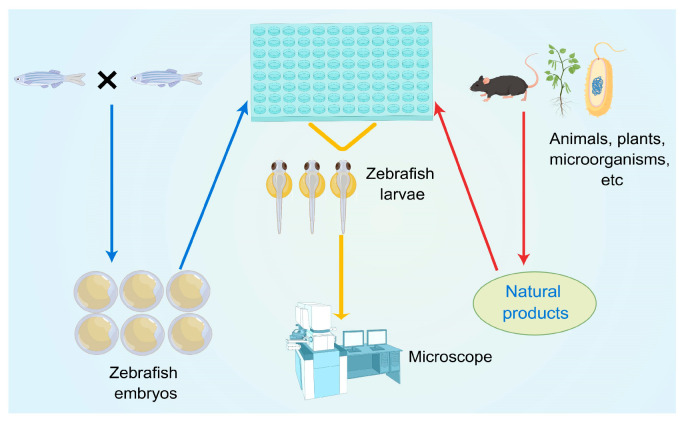
Detection of acute toxicity of natural products in zebrafish models.

**Figure 7:**
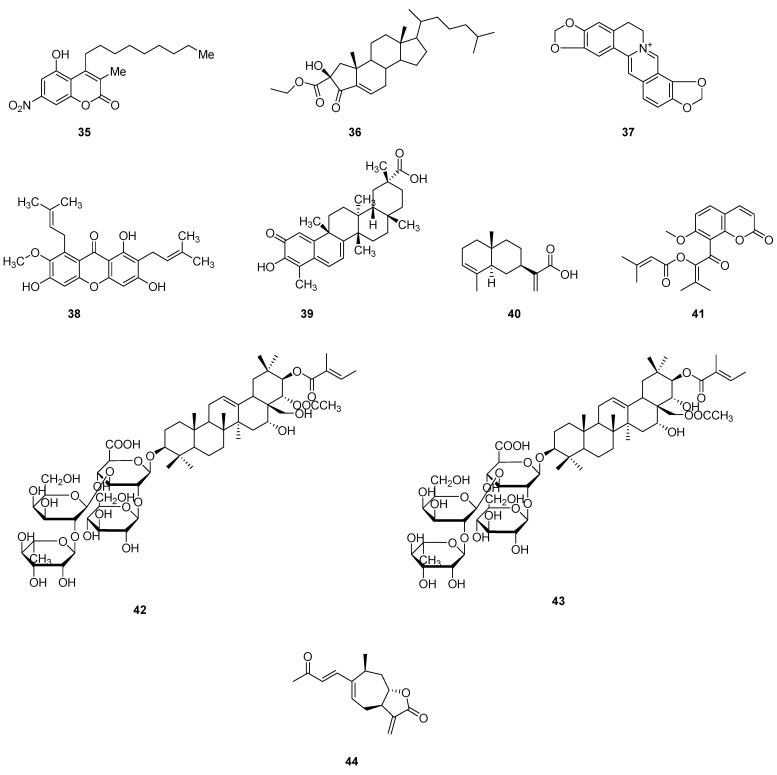
Chemical structures of natural compounds (**35**–**44**) evaluated in zebrafish models.

Zebrafish have been used in a variety of previous studies to determine neurotoxicity, cardiotoxicity, hepatotoxicity, liver toxicity, renal toxicity, developmental toxicity, reproductive toxicity, acute toxicity, etc. [82,84,85]. It should be noted that, while using the zebrafish embryos for the detection of natural product toxicity, not every type of toxicity was accordingly tested. One common test item is acute toxicity, whereas others such as neurotoxicity, cardiac toxicity, and genotoxicity are less tested [83]. The process of detecting the acute toxicity of natural products using the zebrafish embryos is shown in Figure 6. Moreover, some examples of using zebrafish embryos for natural products’ toxicity evaluation are also stated in the following part. Table 4 shows the collection of these compounds/extracts. The structures of some compounds (**35**–**44**) can be found in Figure 7.

Zebrafish models were employed by Liman et al., (2019) to detect the maximum treatment concentration of G-Rh2 (**1**) [36]. They treated zebrafish embryos with different concentrations of G-Rh2 and observed the morphological changes of embryos. The data demonstrate that embryos treated with G-Rh2 up to 120 μM exhibit some morphological defects, such as tail curvature and pericardial oedema, whereas G-Rh2 at 84.5 μM did not exhibit any morphological defects and was chosen as the maximal treatment concentration.

The in vivo toxicity of different concentrations of fucoidan was also investigated using zebrafish models [39]. In this study, Bae et al., (2020) treated 24 hpf zebrafish with fucoidan for 48 h and observed morphological changes under the microscope. It was found that fucoidan (100, 200, and 300 μg/mL) had no significant effect on the development of zebrafish embryos. Moreover, they used the same methods to detect the toxicity of different concentrations of fucosterol in vivo [45]. As a result, fucosterol (40, 60, and 100 μM) had no significant effect on the development of the zebrafish embryos, indicating that fucoidan had a low toxicity and potential as a medication. By the same means, they also evaluated the safety of osthole (**22**) [68]. The results showed that the survival rate of zebrafish embryos treated with different concentrations of osthole was higher than 90% (5, 10, and 20 μM), indicating that osthole has low toxicity towards common cells.

The in vivo toxicity of crude extracts of *Rumex vesicarius* L. was tested by Farooq et al., (2019) using zebrafish models [86]. They treated zebrafish embryos with the extracts (0.001–300 μg/mL) and observed embryos at 24, 48, and 72 hpf. The results demonstrated that zebrafish treated with leaves, stems, roots, and flowers extracts of *R. vesicarius* showed no toxicity, suggesting that the components of *Rumex vesicarius* L. had a good potential for use in patent medicine. Using the same method, Farooq et al., (2020) evaluated the in vivo toxicity of crude extracts of *M. sinaica* [35]. As a result, zebrafish treated with leaves and stem extract resulted in mild cardiac edema, whereas roots and shoot extract did not cause severe distortion to zebrafish, indicating that the roots and shoots extracts of *M. sinaica* had less toxicity and were more amenable to use as medicines.

In addition, the in vivo toxicity of betulinic acid (**26**) was evaluated by Jiao et al., (2019) using zebrafish models [71]. Zebrafish embryos were treated with betulinic acid of different concentrations (10–160 μM). At 24 h, 48 h, 72 h, or 96 h, the morphological changes of these zebrafish were detected. There was no obvious embryotoxicity or teratogenicity during the hatching and development of zebrafish embryos treated with betulinic acid.

Breeta et al., (2018) used zebrafish to verify the toxicity of the leaf extract of *Thuja orientalis* L. in vivo [87]. Zebrafish embryos were treated with leaf extract of different concentrations and were monitored every 24 h from 24 hpf to 144 hpf. The findings indicated that the toxicity of the leaf extract increased in a time-dependent and dose-dependent manner (0.15–2.40 mg/mL), and that 0.6 mg/mL of *T. orientalis* leaf extract is the optimum concentration. The LC_50_ at 96 hpf was 0.7029 mg/mL.

Likewise, Ma et al., (2022) used zebrafish for in vivo toxicity testing of the leaf extract of Miracle berry *S. dulcificum*) [49]. They treated zebrafish with different concentrations of the leaf extract and calculated the LC_50_ of zebrafish at 72 hpf. The results showed that zebrafish did not die when the concentration of the extract was below 25 μg/mL, whereas the LC_50_ from the extract to the zebrafish was found to be 100 μg/mL.

Furthermore, Said et al., (2020) used zebrafish models to test the toxicity of the crude extract of *Tephrosia vogelii* Hook.f. as well as pesticides such as tephrosin, rotenone, and deguelin in vivo [88]. They treated zebrafish with the extracts tephrosin, rotenone, and deguelin, and observed the morphological changes of embryos at 24, 48, and 72 hpf. As can be seen from the results, the crude extract of *T. vogelii* had cytotoxicity and had strong efficacy at low concentrations (at 48 hpf, the mortality rate reached 100% at a concentration of 50 nM), indicating the extract might be used as a natural-based substitute for pesticide components. The LC_50_ for this compound at 48 hpf was 4.8 nM.

Anaya-Eugenio et al., (2020) also used the zebrafish model to preliminarily evaluate the cytotoxicity of JBIR-99 extracted from the fungi *Parengyodontium album* MEXU 30,054 in vivo [89]. They used 50 μM JBIR-99 to treat zebrafish for 24 h, and found that JBIR-99 had no effect on the development of zebrafish and was non-toxic to zebrafish under this concentration.

Myxocoumarin B (**35**) is a natural compound that possesses anti-bacterial activity. Müller et al., (2018) used the zebrafish model to preliminarily evaluate the in vivo cytotoxicity of myxocoumarin B isolated from *stigmatella aurantiaca* MYX-030 [90]. The LC_50_ value of myxocoumarin B in zebrafish models was 344 µM. Moreover, myxocoumarin B has minimal in vivo toxicity and it did not affect zebrafish development at 250 μM.

Rajaram et al., (2018) used the zebrafish model to make a preliminary assessment of the acute toxicity of 2-ethoxycarbonyl-2-β-hydroxy-A-nor-cholest-5-ene-4one (ECHC) (**36**) isolated from *Acropora formosa* in vivo [91]. They exposed zebrafish to 1000 µg/L of ECHC solution and changed the solution every 24 h. The organs of all zebrafish were stained with hematoxylin and eosin. After 21 days, the zebrafish were sacrificed and their organs were dissected and observed. The result showed that these zebrafish had no obvious morphological damage, indicating that ECHC is almost non-toxic to zebrafish.

The in vivo toxicity of *Juglans regia* L. extracts was determined by Rajiv et al., (2021) using the zebrafish models [92]. As could be seen from the experimental results, the LC_50_ value of *J. repens* extracts in zebrafish is 169.2 µg/mL, 30 µg/mL of the extract did not show obvious impact on the growth of zebrafish, 130–380 µg/mL of the extract could result in the growth retardation of zebrafish with underdeveloped embryos, and 560 µg/mL of the extract could cause the death of zebrafish. The LC_50_ listed above was calculated at 72 hpf.

Coptisine (**37**) is one of the *Coptis chinensis* Franch. extracts with an isoquinoline structure. Nakonieczna et al., (2022) used the zebrafish model to test the safety of coptisine isolated from *C. chinensis* [93]. It is found that, at 125 µg/mL, zebrafish did not have morphological abnormalities, but at 187.5 and 250 µg/mL, there were different degrees of abnormalities, such as developmental delay, necrosis of the yolk sac, and so on.

Nugitrangson et al., (2015) used zebrafish models to preliminarily evaluate the toxicity of *α*-mangostin (**38**) purified from Thai stingless bee *(Tetragonula laeviceps)* cerumen in vivo [94]. Zebrafish embryos were treated with different concentrations of *α*-mangostin (3, 6, 9, 12, and 15 µM), and the number of dead embryos was counted. The results showed that the IC_50_ was 9.4 µM at 72 hpf.

Wang et al., (2010) also used the zebrafish model to evaluate the in vivo toxicity of celastrol (**39**) separated from *Tripterygium wilfordii* Hook F. [95]. The results indicated that celastrol with a concentration higher than 1.0 μM would delay the hatching of zebrafish and the media effect concentration (EC_50_) for delayed hatching was 1.02 μM and the IC_50_ was 1.40 µM at 24 hpf.

Moreover, α-costic acid (**40**) is a plant-derived sesquiterpenoid. Sangermano et al., (2021) determined the in vivo toxicity of α-costic acid isolated from *Dittrichia viscosa* (L.) Greuter using zebrafish models [96]. The results demonstrated that 50 µM of α-costic acid results in obvious toxicity to zebrafish. Therefore, α-costic acid is not recommended as a safe naturally occurring pesticide for agricultural application.

Similarly, Carcache et al., (2022) preliminarily evaluated the in vivo toxicity of kimcoungin (**41**) isolated from *Glycosmis ovoidea* Pierre via the zebrafish model [97]. They treated zebrafish embryos with 50 µM kimcoungin and observed whether the embryos were abnormal at 24 hpf, and found that kimcoungin was non-toxic to zebrafish embryos at 50 µM.

To add to the preexisting literature, Bich-Loan et al., (2021) obtained the ethanol extract of *Anisomeles indica* (L.) Kuntze and evaluated the in vivo toxicity with the zebrafish model [98]. As a result, at 75 mg/L, the ethanol extract had a slight impact on zebrafish embryos, including a malformation rate of 5%, a mortality rate of 2.5%, and a hatching efficiency of 70%. At a concentration of 100 mg/L, 90% of zebrafish had developed malformations. When the concentration exceeded 150 mg/L, the embryos died in large numbers.

Yumnamcha et al., (2021) obtained the aqueous extract of *Millettia pachycarpa* Benth. (AEMP) and evaluated its activity in vivo with the zebrafish model [99]. It turned out that different concentrations (1.5, 3, 4.5, 6, or 7.5 µg/mL) of AEMP were toxic to zebrafish embryos in a dose-dependent manner. The LC_50_ at 96 hpf was 4.28 µg/mL.

Further, exopolysaccharides (EPS) are extracellular polysaccharides/polysugars secreted by microorganisms and play roles in cell adhesion and biofilm formation. Usuldin et al., (2021) used the zebrafish model to determine the in vivo toxicity of EPS isolated from mushroom mycelial biomass (*Lignosus rhinocerotis* (Cooke) Ryvarden) [100]. They treated zebrafish embryos with different concentrations of EPS (0.16–10 mg/mL) and recorded the morphological distortion and the number of deaths every 24 h for a total of 120 h. The results showed that the survival rate of zebrafish decreased with the increasing of EPS concentration. Additionally, the survival rate of zebrafish embryos (at 96 hpf) treated with EPS > 1.25 mg/mL was found to be 0%. The LC_50_ at 96 hpf was 0.41 mg/mL.

Nishimura et al., (2021) extracted two triterpene saponin substances, jegosaponin A (**42**) and B (**43**), from *Styrax japonicus* Siebold & Zucc., and evaluated their toxicity in vivo using the zebrafish model [101]. As a result, the LC_50_ of jegosaponin A and B are measured to be 0.5 µM and 1.3 µM at 29 hpf, respectively. Moreover, the toxicity after treatment for 24 h was almost the same as that at 120 h, indicating that the toxicity of jegosaponin A and B to zebrafish occurred within a short period.

In addition, Wnorowski et al., (2020) extracted the major component carlina oxide (chemical name: 2-(3-phenylprop-1-ynyl)furan) from the roots of *Carlina acaulis* L., and evaluated its toxicity in vivo with the zebrafish model [102]. As can be seen from the findings, the LC_50_ of carlina oxide in zebrafish is 10.13 µg/mL at 96 hpf, and the carlina oxide-treated zebrafish showed significant distortion, such as craniofacial malformations, yolk sac edema, and shortened tails.

Likewise, Tan et al., (2018) obtained the crude butanol extract of *Streptomyces californicus* TY004-069 and isolated a macrolide from the butanol extract [103]. They used the zebrafish model to evaluate its toxicity in vivo and found that the development of zebrafish embryos was prevented from 4 to 6 h after 24 h of treatment (20 µM), and all embryos died at 48 hpf.

Yang et al., (2021) preliminarily evaluated thetoxicity of xanthatin (**44**) isolated from *Xanthium spinosum* L. and *Dittrichia graveolens* (L.) in vivo via the zebrafish model [104]. They treated zebrafish with xanthatin of different concentrations (0.31, 0.63, 1.25, 2.50, 5.00, and 10 µM). The results demonstrated that the maximum safe concentration of xanthatin is 5 µM (the survival rate of zebrafish is higher than 80%).

**Table 4:** Application of zebrafish models to detect the toxicity of natural products.

Year	Compound/Extract	Source	Positive Control	Growing Stage of Zebrafish	Results
2019 [86]	leaves, stems, roots, and flowers extract	*Rumex vesicarius* L. (Humeidh)	-	24, 48, and 72 hpf	no toxicity below 300 mg/mL
2020 [35]	leaf, stem, root, and shoot extract	*M. sinaica*	-	72 hpf	Roots and shoots extracts had less toxicity
2018 [87]	leaf extract	*Thuja orientalis* L.	-	96 hpf	LC_50_ = 0.7029 mg/mL
2022 [49]	leaf extract	*S. dulcificum*	-	72 hpf	LC_50_ = 100 μg/mL
2020 [88]	crude extract	*Tephrosia vogelii* Hook.f.	rotenone, deguelin, tephrosin.	48 hpf	LC_50_ = 4.8 nM
2021 [92]	extract	*Jussiaea repens* L.	-	72 hpf	LC_50_ = 169.2 µg/mL
2021 [98]	ethanol extract	*Anisomeles indica* (L.) Kuntze	-	48, 72, and 96 hpf	no toxicity below 75 mg/L
2015 [99]	aqueous extract	*Millettia pachycarpa* Benth.	-	96 hpf	LC_50_ = 4.28 µg/mL
2020 [39]	fucoidan	*F.vesiculosus*	-	48 hpf	no significant effect (100, 200, and 300 μg/mL)
2019 [89]	JBIR-99	*Parengyodontium album* MEXU 30054	cycloheximide	48 hpf	non-toxic (50 μM)
2021 [100]	exopolysaccharides (EPS)	*Lignosus rhinocerotis* (Cooke) Ryvarden	-	96 hpf	LC_50_ = 0.41 mg/mL
2020 [102]	carlina oxide	*Carlina acaulis* L.	Acetone,	96 hpf	LC_50_ = 10.13 µg/mL
2018 [103]	macrolide	*Streptomyces* *californicus* TY004-069	sodium azide (NaN3).	24 hpf	lethal (20 μM)
2021 [36]	ginsenoside Rh2 (**1**)	red ginseng (*P. ginseng*)	-	24 hpf	no toxicity below 84.85 μM
2020 [45]	fucosterol (**9**)	brown algae (*S. fusiforme*)	-	24 hpf	no significant effect (40, 60, and 100 μM)
2020 [68]	osthole (**22**)	*A. archangelica, A. pubescens*, and *C. monnieri*	-	24 hpf	not affecting the survival rate (5,10, and 20 μM)
2019 [71]	betulinic acid (**26**)	birch (*Betula platyphylla* Sukaczev) bark	-	24, 48, 72, and 96 hpf	no obvious embryo toxicity or teratogenicity (10, 20, 40, 80, and 160 μM)
2018 [90]	myxocoumarin B (**35**)	*Stigmatella aurantiaca* MYX-030	-	114 hpf	no toxicity below 250 μM
2018 [91]	2-ethoxycarbonyl-2-β-hydroxy-A-nor-cholest-5-ene-4one (ECHC) (**36**)	*Acropora formosa*	-	21 days	nearly non-toxic (1000 µg/L)
2022 [93]	coptisine (37)	*Coptis chinensis* Franch.	-	96 hpf	no toxicity below 390.24 μM
2015 [94]	α-mangostin (**38**)	Thai stingless bee *(Tetragonula laeviceps)* cerumen	-	72 hpf	LC_50_ = 9.4 µM
2010 [95]	celastrol (**39**)	*Tripterygium wilfordii* Hook F.	-	24 hpf	LC_50_ = 1.40 µM
2021 [96]	α-costic acid (**40**)	*Dittrichia viscosa* (L.) Greuter	-	48 hpf	lethal (50 µM)
2022 [97]	kimcoungin (**41**)	*Glycosmis ovoidea* Pierre	cycloheximide	24 hpf	non-toxic (50 µM)
2021 [101]	jegosaponin A and B (**42**) (**43**)	*Styrax japonicus* Siebold & Zucc.	-	29 hpf	LC_50_ = 0.5 and 1.3 µM, respectively
2021 [104]	xanthatin (**44**)	*Xanthium spinosum* L., *Dittrichia graveolens* L.	-	72 hpf	maximum safe concentration = 5 µM

## 4. Conclusions

Natural products are a major source of anti-cancer drugs. Animal models are indispensable tools for investigating the pathogenesis, pathophysiology, and mechanisms of tumor invasion and metastasis and novel therapeutic approaches to cancer [105]. Zebrafish are time-efficient and cost-effective animal models for rapid assessment of anti-cancer activity and toxicity [106], and have been widely used as practical tools for the development of new drugs. Moreover, zebrafish are good intermediate surrogates prior to more laborious pharmacokinetic studies and applying more costly mammalian models [107]. In this review, we summarized the utilization of zebrafish models to detect anti-cancer activities of natural products over the past few years.

Transgenic zebrafish and zebrafish xenograft models are the most widely used approaches for assessing in vivo anti-cancer activity. The key point for a variety of transgenic models is that these genes can allow zebrafish to express fluorescent proteins in vivo, and the development of blood vessels in zebrafish embryos can be observed very intuitively under the fluorescent microscope, thus making a reasonable evaluation of the anti-angiogenic activity of natural products. The zebrafish xenograft model technology has gradually matured in recent years, although the mouse xenotransplantation model is still the “gold standard”. The key advantage of zebrafish embryo xenograft model is its underdeveloped immune system in the embryonic stage, which could greatly diminish the immune rejection response during the transplantation of dye-labeled human cancer cells. Importantly, fluorescence microscopy could be used to directly observe the development of human cancer cell zebrafish xenograft models, and, subsequently, visual evaluation can be performed to test for the in vivo anti-cancer effect or toxicity of natural products. This method has been used for drug-like compound screening, water quality inspection, ecosystem evaluations, and so on. However, up to now, researchers mainly used zebrafish models to test the acute toxicity of natural products by observing the morphological change (e.g., distortion and growth retardation) and death of zebrafish. However, other toxicities such as neurotoxicity, cardiac toxicity, and genotoxicity also need to be extensively tested. Notably, there are rarely any studies about comparative pharmacology on natural products with similar structures or pharmacophores. Moreover, the quantitative structure–activity relationship (QSAR) of anti-cancer natural products in the zebrafish models needs to be deeply investigated.

During the past few decades, the pharmacological research on anti-cancer activity and drug toxicity evaluation of natural products had been successfully conducted on traditional cancer cell lines and cancer cell rodent xenograft models. In the newly developed zebrafish models, the related pharmacological research occurs only at the very early stages [108,109]. Nowadays, the emergence of vascular(site)-specific gene transfer, the development of various human cancer zebrafish xenograft models, new fluorescent bioimaging probes, as well as high-resolution microscopy instruments and technologies are expanding the capability of zebrafish models for natural products evaluation. We anticipate that the zebrafish model will gradually be developed as an economically-viable, efficient, convenient, and promising organism for the innovation of novel natural-based drugs in the future.

## Data Availability

Data sharing not applicable.

## References

[1] Siegel R.L., Miller K.D., Fuchs H.E., Jemal A. (2022). Cancer statistics, 2022. CA Cancer J. Clin..

[2] Xia C., Dong X., Li H., Cao M., Sun D., He S., Yang F., Yan X., Zhang S., Li N. (2022). Cancer statistics in China and United States, 2022: Profiles, trends, and determinants. Chin. Med. J..

[3] Khalifa S.A.M., Elias N., Farag M.A., Chen L., Saeed A., Hegazy M.F., Moustafa M.S., Abd El-Wahed A., Al-Mousawi S.M., Musharraf S.G. (2019). Marine Natural Products: A Source of Novel Anti-cancer Drugs. Mar. Drugs.

[4] Guo M., Jin J., Zhao D., Rong Z., Cao L.Q., Li A.H., Sun X.Y., Jia L.Y., Wang Y.D., Huang L. (2022). Research Advances on Anti-Cancer Natural Products. Front. Oncol..

[5] Dutta S., Mahalanobish S., Saha S., Ghosh S., Sil P.C. (2019). Natural products: An upcoming therapeutic approach to cancer. Food Chem. Toxicol..

[6] Ma L., Zhang M., Zhao R., Wang D., Ma Y., Li A. (2021). Plant Natural Products: Promising Resources for Cancer Chemoprevention. Molecules.

[7] Kumar A., Jaitak V. (2019). Natural products as multidrug resistance modulators in cancer. Eur. J. Med. Chem..

[8] Newman D.J., Cragg G.M. (2020). Natural Products as Sources of New Drugs over the Nearly Four Decades from 01/1981 to 09/2019. J. Nat. Prod..

[9] Fu Y., Luo J., Qin J., Yang M. (2019). Screening techniques for the identification of bioactive compounds in natural products. J. Pharm. Biomed. Anal..

[10] Howe K., Clark M.D., Torroja C.F., Torrance J., Berthelot C., Muffato M., Collins J.E., Humphray S., McLaren K., Matthews L. (2013). The zebrafish reference genome sequence and its relationship to the human genome. Nature.

[11] Patton E.E., Zon L.I., Langenau D.M. (2021). Zebrafish disease models in drug discovery: From preclinical modelling to clinical trials. Nat. Rev. Drug Discov..

[12] Brown H.K., Schiavone K., Tazzyman S., Heymann D., Chico T.J. (2017). Zebrafish xenograft models of cancer and metastasis for drug discovery. Expert Opin. Drug Discov..

[13] Lam P.Y., Peterson R.T. (2019). Developing zebrafish disease models for in vivo small molecule screens. Curr. Opin. Chem. Biol..

[14] Lubin A., Otterstrom J., Hoade Y., Bjedov I., Stead E., Whelan M., Gestri G., Paran Y., Payne E. (2021). A versatile, automated and high-throughput drug screening platform for zebrafish embryos. Biol. Open.

[15] Santoro M.M. (2014). Antiangiogenic cancer drug using the zebrafish model. Arterioscler. Thromb. Vasc. Biol..

[16] Cascallar M., Alijas S., Pensado-Lopez A., Vazquez-Rios A.J., Sanchez L., Pineiro R., de la Fuente M. (2022). What Zebrafish and Nanotechnology Can Offer for Cancer Treatments in the Age of Personalized Medicine. Cancers.

[17] Al-Thani H.F., Shurbaji S., Yalcin H.C. (2021). Zebrafish as a Model for Anti-cancer Nanomedicine Studies. Pharmaceuticals.

[18] Zon L.I., Le X. (2008). Potential of zebrafish for anti-cancer drug screening. Expert Opin. Drug Discov..

[19] Shive H.R. (2012). Zebrafish Models for Human Cancer. Vet. Pathol..

[20] Kirchberger S., Sturtzel C., Pascoal S., Distel M. (2017). Quo natas, Danio?—Recent Progress in Modeling Cancer in Zebrafish. Front. Oncol..

[21] Lin F.J., Li H., Wu D.T., Zhuang Q.G., Li H.B., Geng F., Gan R.Y. (2022). Recent development in zebrafish model for bioactivity and safety evaluation of natural products. Crit. Rev. Food Sci. Nutr..

[22] Bunton T.E. (1996). Experimental chemical carcinogenesis in fish. Toxicol. Pathol..

[23] Spitsbergen J.M., Tsai H.-W., Reddy A., Miller T., Arbogast D., Hendricks J.D., Bailey G. (2000). Neoplasia in Zebrafish (*Danio rerio*) Treated with N-methyl-N’nitro-N-nitrosoguanidine by Three Exposure Routes at different Developmental Stages. Toxicol. Pathol..

[24] Spitsbergen J.M., Tsai H.-W., Reddy A., Miller T., Arbogast D., Hendricks J.D., Bailey G. (2000). Neoplasia in zebrafish (*Danio rerio*) treated with 7, 12-Diniethylbenz [a] anthracene by two exposure routes at different developmental stages. Toxicol. Pathol..

[25] Beckwith L.G., Moore J.L., Tsao-Wu G.S., Harshbarger J.C., Cheng K.C. (2000). Ethylnitrosourea induces neoplasia in zebrafish (*Danio rerio*). Lab. Investig..

[26] Mizgireuv I.V., Majorova I.G., Gorodinskaya V.M., Khudoley V.V., Revskoy S.Y. (2004). Carcinogenic effect of N-nitrosodimethylamine on diploid and triploid zebrafish (*Danio rerio*). Toxicol. Pathol..

[27] Kawasaki T., Shimizu Y. (2021). Carcinogenesis Models Using Small Fish. Chem. Pharm. Bull..

[28] Mimeault M., Batra S.K. (2013). Emergence of zebrafish models in oncology for validating novel anti-cancer drug targets and nanomaterials. Drug Discov. Today.

[29] Hsu S.Y., Wen Z.H., Shih P.C., Kuo H.M., Lin S.C., Liu H.T., Lee Y.H., Wang Y.J., Chen W.F., Chen N.F. (2022). Sinularin Induces Oxidative Stress-Mediated Apoptosis and Mitochondrial Dysfunction, and Inhibits Angiogenesis in Glioblastoma Cells. Antioxidants.

[30] Liang F., Han Y., Gao H., Xin S., Chen S., Wang N., Qin W., Zhong H., Lin S., Yao X. (2015). Kaempferol Identified by Zebrafish Assay and Fine Fractionations Strategy from *Dysosma versipellis* Inhibits Angiogenesis through VEGF and FGF Pathways. Sci. Rep..

[31] Jiang X., Zhou J., Lin Q., Gong G., Sun H., Liu W., Guo Q., Feng F., Qu W. (2018). Anti-angiogenic and anti-cancer effects of baicalein derivatives based on transgenic zebrafish model. Bioorg. Med. Chem..

[32] Rafaella d.S.B., Canedo P.A., Davi F., Lopes R.T. (2022). Transgenic zebrafish (*Danio rerio*) as an emerging model system in ecotoxicology and toxicology: Historical review, recent advances, and trends. Sci. Total Environ..

[33] Choe C.P., Choi S.Y., Kee Y., Kim M.J., Kim S.H., Lee Y., Park H.C., Ro H. (2021). Transgenic fluorescent zebrafish lines that have revolutionized biomedical research. Lab. Anim. Res..

[34] Wang Y., Wang B., Guerram M., Sun L., Shi W., Tian C., Zhu X., Jiang Z., Zhang L. (2015). Deoxypodophyllotoxin suppresses tumor vasculature in HUVECs by promoting cytoskeleton remodeling through LKB1-AMPK dependent Rho A activation. Oncotarget.

[35] Farooq M., Nasr F.A., Almoutiri N.D., Al-yahya N., Wadaan M.A., Abutaha N. (2020). The phytochemical screening and antiangiogenic activity of audthan al himar (*Moricandia sinaica Boiss.*) extracts in zebrafish embryos and human umbilical vein endothelial cells. J. King Saud Univ.-Sci..

[36] Liman M.A., Yongxiao Q.I., Wenji W., Qianyi Z. (2020). Anti-angiogenic Effect of Ginsenoside Rh2 by Downregulation of VEGF in a Zebrafish Model. Pak. J. Zool..

[37] Long W., Wang M., Luo X., Huang G., Chen J. (2018). Murrangatin suppresses angiogenesis induced by tumor cell-derived media and inhibits AKT activation in zebrafish and endothelial cells. Drug Des. Devel. Ther..

[38] Lam K.H., Alex D., Lam I.K., Tsui S.K., Yang Z.F., Lee S.M. (2011). Nobiletin, a polymethoxylated flavonoid from citrus, shows anti-angiogenic activity in a zebrafish in vivo model and HUVEC in vitro model. J. Cell Biochem..

[39] Bae H., Lee J.Y., Yang C., Song G., Lim W. (2020). Fucoidan Derived from Fucus vesiculosus Inhibits the Development of Human Ovarian Cancer via the Disturbance of Calcium Homeostasis, Endoplasmic Reticulum Stress, and Angiogenesis. Mar. Drugs.

[40] Hsu W.J., Lin M.H., Kuo T.C., Chou C.M., Mi F.L., Cheng C.H., Lin C.W. (2020). Fucoidan from Laminaria japonica exerts antitumor effects on angiogenesis and micrometastasis in triple-negative breast cancer cells. Int. J. Biol. Macromol..

[41] Pan C.C., Shah N., Kumar S., Wheeler S.E., Cinti J., Hoyt D.G., Beattie C.E., An M., Mythreye K., Rakotondraibe L.H. (2017). Angiostatic actions of capsicodendrin through selective inhibition of VEGFR2-mediated AKT signaling and disregulated autophagy. Oncotarget.

[42] Hsi H.Y., Wang S.W., Cheng C.H., Pang K.L., Leu J.Y., Chang S.H., Lee Y.T., Kuo Y.H., Huang C.Y., Lee T.H. (2022). Chemical Constituents and Anti-Angiogenic Principles from a Marine Algicolous *Penicillium sumatraense* SC29. Molecules.

[43] Zhao Y., Zhang X., Li Y., Li Y., Zhang H., Song Z., Xu J., Guo Y. (2022). A natural xanthone suppresses lung cancer growth and metastasis by targeting STAT3 and FAK signaling pathways. Phytomedicine.

[44] Zhao C., Kam H.T., Chen Y., Gong G., Hoi M.P., Skalicka-Wozniak K., Dias A.C.P., Lee S.M. (2021). Crocetin and Its Glycoside Crocin, Two Bioactive Constituents from *Crocus sativus* L. (Saffron), Differentially Inhibit Angiogenesis by Inhibiting Endothelial Cytoskeleton Organization and Cell Migration Through VEGFR2/SRC/FAK and VEGFR2/MEK/ERK Signaling Pathways. Front. Pharmacol..

[45] Bae H., Lee J.Y., Song G., Lim W. (2020). Fucosterol Suppresses the Progression of Human Ovarian Cancer by Inducing Mitochondrial Dysfunction and Endoplasmic Reticulum Stress. Mar. Drugs.

[46] Deshmukh D., Hsu Y.F., Chiu C.C., Jadhao M., Hsu S.C.N., Hu S.Y., Yang S.H., Liu W. (2023). Antiangiogenic potential of Lepista nuda extract suppressing MAPK/p38 signaling-mediated developmental angiogenesis in zebrafish and HUVECs. Biomed. Pharm..

[47] Li Y., Wang H., Liu W., Hou J., Xu J., Guo Y., Hu P. (2022). Cratoxylumxanthone C, a natural xanthone, inhibits lung cancer proliferation and metastasis by regulating STAT3 and FAK signal pathways. Front. Pharmacol..

[48] Lee J.Y., Bae H., Yang C., Park S., Youn B.S., Kim H.S., Song G., Lim W. (2020). Eupatilin Promotes Cell Death by Calcium Influx through ER-Mitochondria Axis with SERPINB11 Inhibition in Epithelial Ovarian Cancer. Cancers.

[49] Ma F.Y., Zhang X.M., Li Y., Zhang M., Tu X.H., Du L.Q. (2022). Identification of phenolics from miracle berry (Synsepalum dulcificum) leaf extract and its antiangiogenesis and anti-cancer activities. Front. Nutr..

[50] Li Q., Wang X., Dai T., Liu C., Li T., McClements D.J., Chen J., Liu J. (2016). Proanthocyanidins, Isolated from Choerospondias axillaris Fruit Peels, Exhibit Potent Antioxidant Activities in Vitro and a Novel Anti-angiogenic Property in Vitro and in Vivo. J. Agric. Food Chem..

[51] Garkavtsev I., Chauhan V.P., Wong H.K., Mukhopadhyay A., Glicksman M.A., Peterson R.T., Jain R.K. (2011). Dehydro-alpha-lapachone, a plant product with antivascular activity. Proc. Natl. Acad. Sci. USA.

[52] Li P., Yue G.G., Kwok H.F., Long C.L., Lau C.B., Kennelly E.J. (2017). Using Ultra-Performance Liquid Chromatography Quadrupole Time of Flight Mass Spectrometry-Based Chemometrics for the Identification of Anti-angiogenic Biflavonoids from Edible Garcinia Species. J. Agric. Food Chem..

[53] Yue G.G.-L., Kwok H.-F., Lee J.K.-M., Jiang L., Chan K.-M., Cheng L., Wong E.C.-W., Leung P.-C., Fung K.-P., Lau C.B.-S. (2015). Novel anti-angiogenic effects of aromatic-turmerone, essential oil isolated from spice turmeric. J. Funct. Foods.

[54] Kim N.H., Pham N.B., Quinn R.J., Shim J.S., Cho H., Cho S.M., Park S.W., Kim J.H., Seok S.H., Oh J.W. (2015). The Small Molecule R-(-)-beta-O-Methylsynephrine Binds to Nucleoporin 153 kDa and Inhibits Angiogenesis. Int. J. Biol. Sci..

[55] Chen K., Fan Y., Gu J., Han Z., Zeng H., Mao C., Wang C. (2020). In vivo Screening of Natural Products Against Angiogenesis and Mechanisms of Anti-Angiogenic Activity of Deoxysappanone B 7,4′-Dimethyl Ether. Drug Des. Devel. Ther..

[56] Zhou Z.Y., Zhao W.R., Xiao Y., Zhou X.M., Huang C., Shi W.T., Zhang J., Ye Q., Chen X.L., Tang J.Y. (2020). Antiangiogenesis effect of timosaponin AIII on HUVECs in vitro and zebrafish embryos in vivo. Acta Pharmacol. Sin..

[57] Chen K., Wang C., Fan Y., Gu J., Han Z., Wang Y., Gao L., Zeng H. (2018). Identification of mundoserone by zebrafish in vivo screening as a natural product with anti-angiogenic activity. Exp. Ther. Med..

[58] Hu J., Lin S., Huang J.J., Cheung P.C.K. (2018). Mechanistic Study of the In Vitro and In Vivo Inhibitory Effects of Protocatechuic Acid and Syringic Acid on VEGF-Induced Angiogenesis. J. Agric. Food Chem..

[59] Zhang W., Liu B., Feng Y., Liu J., Ma Z., Zheng J., Xia Q., Ni Y., Li F., Lin R. (2017). Anti-angiogenic activity of water extract from Euphorbia pekinensis Rupr. J. Ethnopharmacol..

[60] Zhong T., Piao L., Kim H.J., Liu X., Jiang S., Liu G. (2017). Chlorogenic Acid-Enriched Extract of Ilex kudingcha C.J. Tseng Inhibits Angiogenesis in Zebrafish. J. Med. Food.

[61] Zhang B., Xuan C., Ji Y., Zhang W., Wang D. (2015). Zebrafish xenotransplantation as a tool for in vivo cancer study. Fam. Cancer.

[62] Wertman J., Veinotte C.J., Dellaire G., Berman J.N. (2016). The Zebrafish Xenograft Platform: Evolution of a Novel Cancer Model and Preclinical Screening Tool. Adv. Exp. Med. Biol..

[63] Lee L.M.J., Seftor E.A., Bonde G., Cornell R.A., Hendrix M.J.C. (2005). The fate of human malignant melanoma cells transplanted into zebrafish embryos: Assessment of migration and cell division in the absence of tumor formation. Dev. Dyn..

[64] Xiao J., Glasgow E., Agarwal S. (2020). Zebrafish Xenografts for Drug Discovery and Personalized Medicine. Trends Cancer.

[65] Konantz M., Balci T.B., Hartwig U.F., Dellaire G., Andre M.C., Berman J.N., Lengerke C. (2012). Zebrafish xenografts as a tool for in vivo studies on human cancer. Ann. N. Y. Acad. Sci..

[66] Barriuso J., Nagaraju R., Hurlstone A. (2015). Zebrafish: A new companion for translational research in oncology. Clin. Cancer Res..

[67] Schneider N.F.Z., Cerella C., Lee J.Y., Mazumder A., Kim K.R., de Carvalho A., Munkert J., Padua R.M., Kreis W., Kim K.W. (2018). Cardiac Glycoside Glucoevatromonoside Induces Cancer Type-Specific Cell Death. Front. Pharmacol..

[68] Bae H., Lee J.Y., Song J., Song G., Lim W. (2021). Osthole interacts with an ER-mitochondria axis and facilitates tumor suppression in ovarian cancer. J. Cell. Physiol..

[69] McKeown B.T., Relja N.J., Hall S.R., Gebremeskel S., MacLeod J.M., Veinotte C.J., Bennett L.G., Ohlund L.B., Sleno L., Jakeman D.L. (2022). Pilot study of jadomycin B pharmacokinetics and anti-tumoral effects in zebrafish larvae and mouse breast cancer xenograft models. Can. J. Physiol. Pharmacol..

[70] Wu X.X., Yue G.G., Dong J.R., Lam C.W., Wong C.K., Qiu M.H., Lau C.B. (2018). Actein Inhibits the Proliferation and Adhesion of Human Breast Cancer Cells and Suppresses Migration in vivo. Front. Pharmacol..

[71] Jiao L., Wang S., Zheng Y., Wang N., Yang B., Wang D., Yang D., Mei W., Zhao Z., Wang Z. (2019). Betulinic acid suppresses breast cancer aerobic glycolysis via caveolin-1/NF-kappaB/c-Myc pathway. Biochem. Pharmacol..

[72] Tian L., Xie K., Sheng D., Wan X., Zhu G. (2017). Antiangiogenic effects of oridonin. BMC Complement. Altern. Med..

[73] Zhu X.Y., Guo D.W., Lao Q.C., Xu Y.Q., Meng Z.K., Xia B., Yang H., Li C.Q., Li P. (2019). Sensitization and synergistic anti-cancer effects of Furanodiene identified in zebrafish models. Sci. Rep..

[74] Kim E.A., Lee J.H., Heo S.J., Jeon Y.J. (2021). Saringosterol acetate isolated from Hizikia fusiforme, an edible brown alga, suppressed hepatocellular carcinoma growth and metastasis in a zebrafish xenograft model. Chem. Biol. Interact..

[75] Zhang L., Yan B., Meng S., Zhou L., Xu Y., Du W., Shan L. (2020). Theaflavin Induces Apoptosis of A375 Human Melanoma Cells and Inhibits Tumor Growth in Xenograft Zebrafishes Through P53- and JNK-Related Mechanism. Front. Pharmacol..

[76] Cao H.H., Liu D.Y., Lai Y.C., Chen Y.Y., Yu L.Z., Shao M., Liu J.S. (2020). Inhibition of the STAT3 Signaling Pathway Contributes to the Anti-Melanoma Activities of Shikonin. Front. Pharmacol..

[77] Chen S.-M., Feng J.-N., Zhao C.-K., Yao L.-C., Wang L.-X., Meng L., Cai S.-Q., Liu C.-Y., Qu L.-K., Jia Y.-X. (2022). A multi-targeting natural product, aiphanol, inhibits tumor growth and metastasis. Am. J. Cancer Res..

[78] Lin Q.H., Qu W., Xu J., Feng F., He M.F. (2018). 1-Methoxycarbony-beta-carboline from Picrasma quassioides exerts anti-angiogenic properties in HUVECs in vitro and zebrafish embryos in vivo. Chin. J. Nat. Med..

[79] Jin W., Zhou L., Yan B., Yan L., Liu F., Tong P., Yu W., Dong X., Xie L., Zhang J. (2018). Theabrownin triggers DNA damage to suppress human osteosarcoma U2OS cells by activating p53 signalling pathway. J. Cell. Mol. Med..

[80] Yu J., Zhong B., Jin L., Hou Y., Ai N., Ge W., Li L., Liu S., Lu J.-J., Chen X. (2020). 2-Methoxy-6-acetyl-7-methyljuglone (MAM) induced programmed necrosis in glioblastoma by targeting NAD(P)H: Quinone oxidoreductase 1 (NQO1). Free. Radic. Biol. Med..

[81] Eimon P.M., Rubinstein A.L. (2009). The use of in vivo zebrafish assays in drug toxicity screening. Expert Opin. Drug Metab. Toxicol..

[82] He J.H., Gao J.M., Huang C.J., Li C.Q. (2014). Zebrafish models for assessing developmental and reproductive toxicity. Neurotoxicol. Teratol..

[83] Miyawaki I. (2020). Application of zebrafish to safety evaluation in drug discovery. J. Toxicol. Pathol..

[84] Lai K.P., Gong Z., Tse W.K.F. (2021). Zebrafish as the toxicant screening model: Transgenic and omics approaches. Aquat. Toxicol..

[85] Zhang Y., Xia Q., Wang J., Zhuang K., Jin H., Liu K. (2022). Progress in using zebrafish as a toxicological model for traditional Chinese medicine. J. Ethnopharmacol..

[86] Farooq M., Abutaha N., Mahboob S., Baabbad A., Almoutiri N.D., Wadaan M. (2020). Investigating the antiangiogenic potential of *Rumex vesicarius* (humeidh), anti-cancer activity in cancer cell lines and assessment of developmental toxicity in zebrafish embryos. Saudi J. Biol. Sci..

[87] Breeta R.E., Jesubatham P.D., Grace V.M.B., Viswanathan S. (2018). Srividya Non-toxic and non teratogenic extract of *Thuja orientalis* L. inhibited angiogenesis in zebra fish and suppressed the growth of human lung cancer cell line. Biomed. Pharmacother..

[88] Said A.H., Solhaug A., Sandvik M., Msuya F.E., Kyewalyanga M.S., Mmochi A.J., Lyche J.L., Hurem S. (2020). Isolation of the Tephrosia vogelii extract and rotenoids and their toxicity in the RTgill-W1 trout cell line and in zebrafish embryos. Toxicon.

[89] Anaya-Eugenio G.D., Rebollar-Ramos D., Gonzalez M.D.C., Raja H., Mata R., Carcache de Blanco E.J. (2019). Apoptotic activity of xanthoquinodin JBIR-99, from Parengyodontium album MEXU 30054, in PC-3 human prostate cancer cells. Chem. Biol. Interact..

[90] Muller J.I., Kusserow K., Hertrampf G., Pavic A., Nikodinovic-Runic J., Gulder T.A.M. (2019). Synthesis and initial biological evaluation of myxocoumarin B. Org. Biomol. Chem..

[91] Ramalingam V., Rajaram R. (2018). 2-Ethoxycarbonyl-2-beta-hydroxy-a-nor-cholest-5-ene-4one: Extraction, structural characterization, antimicrobial, antioxidant, anti-cancer and acute toxicity studies. Steroids.

[92] Rajiv C., Roy S.S., Tamreihao K., Kshetri P., Singh T.S., Sanjita Devi H., Sharma S.K., Ansari M.A., Devi E.D., Devi A.K. (2021). Anticarcinogenic and Antioxidant Action of an Edible Aquatic Flora *Jussiaea repens* L. Using In Vitro Bioassays and In Vivo Zebrafish Model. Molecules.

[93] Nakonieczna S., Grabarska A., Gawel K., Wroblewska-Luczka P., Czerwonka A., Stepulak A., Kukula-Koch W. (2022). Isoquinoline Alkaloids from Coptis chinensis Franch: Focus on Coptisine as a Potential Therapeutic Candidate against Gastric Cancer Cells. Int. J. Mol. Sci..

[94] Nugitrangson P., Puthong S., Iempridee T., Pimtong W., Pornpakakul S., Chanchao C. (2016). In vitro and in vivo characterization of the anti-cancer activity of Thai stingless bee (*Tetragonula laeviceps*) cerumen. Exp. Biol. Med..

[95] Wang S., Liu K., Wang X., He Q., Chen X. (2011). Toxic effects of celastrol on embryonic development of zebrafish (*Danio rerio*). Drug Chem. Toxicol..

[96] Sangermano F., Masi M., Kumar A., Peravali R., Tuzi A., Cimmino A., Vallone D., Giamundo G., Conte I., Evidente A. (2021). In Vitro and In Vivo Toxicity Evaluation of Natural Products with Potential Applications as Biopesticides. Toxins.

[97] Blanco Carcache P.J., Anaya Eugenio G.D., Ninh T.N., Moore C.E., Rivera-Chavez J., Ren Y., Soejarto D.D., Kinghorn A.D. (2022). Cytotoxic constituents of *Glycosmis ovoidea* collected in Vietnam. Fitoterapia.

[98] Bich-Loan N.T., Kien K.T., Thanh N.L., Kim-Thanh N.T., Huy N.Q., The-Hai P., Muller M., Nachtergael A., Duez P., Thang N.D. (2021). Toxicity and Anti-Proliferative Properties of Anisomeles indica Ethanol Extract on Cervical Cancer HeLa Cells and Zebrafish Embryos. Life.

[99] Yumnamcha T., Roy D., Devi M.D., Nongthomba U. (2015). Evaluation of developmental toxicity and apoptotic induction of the aqueous extract of *Millettia pachycarpa* using zebrafish as model organism. Toxicol. Environ. Chem..

[100] Usuldin S.R.A., Wan-Mohtar W.A.A.Q.I., Ilham Z., Jamaludin A.A., Abdullah N.R., Rowan N. (2021). In vivo toxicity of bioreactor-grown biomass and exopolysaccharides from Malaysian tiger milk mushroom mycelium for potential future health applications. Sci. Rep..

[101] Nishimura M., Fuchino H., Takayanagi K., Kawakami H., Nakayama H., Kawahara N., Shimada Y. (2021). Toxicity of Jegosaponins A and B from *Styrax japonica* Siebold et al. Zuccarini in Prostate Cancer Cells and Zebrafish Embryos Resulting from Increased Membrane Permeability. Int. J. Mol. Sci..

[102] Wnorowski A., Wnorowska S., Wojas-Krawczyk K., Grenda A., Staniak M., Michalak A., Wozniak S., Matosiuk D., Biala G., Wojciak M. (2020). Toxicity of Carlina Oxide-A Natural Polyacetylene from the *Carlina acaulis* Roots-In Vitro and In Vivo Study. Toxins.

[103] Tan P.J., Lau B.F., Krishnasamy G., Ng M.F., Husin L.S., Ruslan N., Song D.S.S., Velaithan V., Okuda K.S., Patel V. (2018). Zebrafish embryonic development-interfering macrolides from Streptomyces californicus impact growth and mitochondrial function in human colorectal cancer cells. Process Biochem..

[104] Yang J., Li Y., Zong C., Zhang Q., Ge S., Ma L., Fan J., Zhang J., Jia R. (2021). Xanthatin Selectively Targets Retinoblastoma by Inhibiting the PLK1-Mediated Cell Cycle. Investig. Ophthalmol. Vis. Sci..

[105] Vitale G., Gaudenzi G., Circelli L., Manzoni M.F., Bassi A., Fioritti N., Faggiano A., Colao A., Group N. (2017). Animal models of medullary thyroid cancer: State of the art and view to the future. Endocr.-Relat. Cancer.

[106] Al-Hamaly M.A., Turner L.T., Rivera-Martinez A., Rodriguez A., Blackburn J.S. (2023). Zebrafish Cancer Avatars: A Translational Platform for Analyzing Tumor Heterogeneity and Predicting Patient Outcomes. Int. J. Mol. Sci..

[107] Lee H.C., Lin C.Y., Tsai H.J. (2021). Zebrafish, an In Vivo Platform to Screen Drugs and Proteins for Biomedical Use. Pharmaceuticals.

[108] Nathan J., Kannan R.R. (2020). Antiangiogenic molecules from marine actinomycetes and the importance of using zebrafish model in cancer research. Heliyon.

[109] Okuda K.S., Lee H.M., Velaithan V., Ng M.F., Patel V. (2016). Utilizing Zebrafish to Identify Anti-(Lymph)Angiogenic Compounds for Cancer Treatment: Promise and Future Challenges. Microcirculation.

